# Mitochondria transplantation between living cells

**DOI:** 10.1371/journal.pbio.3001576

**Published:** 2022-03-23

**Authors:** Christoph G. Gäbelein, Qian Feng, Edin Sarajlic, Tomaso Zambelli, Orane Guillaume-Gentil, Benoît Kornmann, Julia A. Vorholt

**Affiliations:** 1 Institute of Microbiology, ETH Zurich, Zurich, Switzerland; 2 Institute of Biochemistry, ETH Zurich, Zurich, Switzerland; 3 SmartTip BV, Enschede, the Netherlands; 4 Institute for Biomedical Engineering, ETH Zurich, Zurich, Switzerland; 5 Department of Biochemistry, University of Oxford, Oxford, United Kingdom; Newcastle University, UNITED KINGDOM

## Abstract

Mitochondria and the complex endomembrane system are hallmarks of eukaryotic cells. To date, it has been difficult to manipulate organelle structures within single live cells. We developed a FluidFM-based approach to extract, inject, and transplant organelles from and into living cells with subcellular spatial resolution. The technology combines atomic force microscopy, optical microscopy, and nanofluidics to achieve force and volume control with real-time inspection. We developed dedicated probes that allow minimally invasive entry into cells and optimized fluid flow to extract specific organelles. When extracting single or a defined number of mitochondria, their morphology transforms into a pearls-on-a-string phenotype due to locally applied fluidic forces. We show that the induced transition is calcium independent and results in isolated, intact mitochondria. Upon cell-to-cell transplantation, the transferred mitochondria fuse to the host cells mitochondrial network. Transplantation of healthy and drug-impaired mitochondria into primary keratinocytes allowed monitoring of mitochondrial subpopulation rescue. Fusion with the mitochondrial network of recipient cells occurred 20 minutes after transplantation and continued for over 16 hours. After transfer of mitochondria and cell propagation over generations, donor mitochondrial DNA (mtDNA) was replicated in recipient cells without the need for selection pressure. The approach opens new prospects for the study of organelle physiology and homeostasis, but also for therapy, mechanobiology, and synthetic biology.

## Abstract 2

Mitochondria and the complex endomembrane system are hallmarks of eukaryotic cells, but it has proved difficult to manipulate organelle structures within single live cells. This study describes a novel microfluidic device that allows the extraction of organelles, including mitochondria, from viable cells and their reintroduction into recipient host cells.

## Introduction

Single-cell surgery approaches promise minimally invasive perturbation, i.e., removal or introduction of cellular compartments without compromising cell viability. Manipulation of mitochondria receives special emphasis due to their central cellular role: They are at the heart of energy conversion and link cellular metabolism to signaling pathways and cell fate decision [1–3]. Mitochondria harbor their own genetic content (mitochondrial DNA, mtDNA), which is prone to accumulating erroneous, disease-causing mutations [4–6], and are subject to quality control [7,8]. Although mitochondria are generally inherited strictly vertically to daughter cells, exchange of larger cellular components including mitochondria has also been observed in tissues of multicellular organisms [9–12]. To reconstitute such transfer events, therapy approaches involve the grafting of purified mitochondria into a damaged area of a tissue or their intravenous injection [13]. However, the fate of these mitochondria is unknown [14].

To date, no suitable technical tools exit to directly study the impact of organelle transmission between individual cells. Such experiments require a technology that enables both extraction and injection of femtoliter to picoliter volumes from and into individual cells. In addition, the investigation of both short- and long-term effects of organelle transplantation requires high transfer efficiency and cell viability throughout the process. Manipulation of interconnected endomembrane structures is particularly challenging, even more than the injection and extraction of small molecules because their handling is more invasive and thus prone to induce irreversible cellular damage.

Miniaturized probes have the potential to manipulate and sample individual cells within their microenvironment at high spatiotemporal resolution [15]. Micropipette-based technologies enable extraction of molecules and single mitochondria via dielectrophoretic trapping [16] or electrowetting [17], showcasing that removal of individual organelle structures is possible without compromising cell viability. Targeted injection of undefined amounts of isolated mitochondria has been achieved using an engineered micropipette-based probe termed the “photothermal nanoblade” [18,19]. The method enables a mitochondrial transfer efficiency of 2%, into mtDNA eliminated ρ0 cells. Following clonal selection, the approach allowed the study of cell populations with alternated mtDNA genotypes [19]. In addition to methods offering single-cell resolution, bulk approaches for the delivery of mitochondria and cytoplasmic content exist, including mitocyoplast cell fusion [20], nonspecific mitochondrial uptake [9,21], and a pressurized device for bulk insertion [22].

Despite substantial progress, established platforms focus on either extraction or injection and have insufficient efficiencies and scalability of volumes; they also focus on indefinitely expandable cancer cell lines. Therefore, extraction and injection of varying amounts of organelles from single cells has not been possible to date, and the response of single cells to transferred mitochondria remains to be explored.

**Fig 1:**
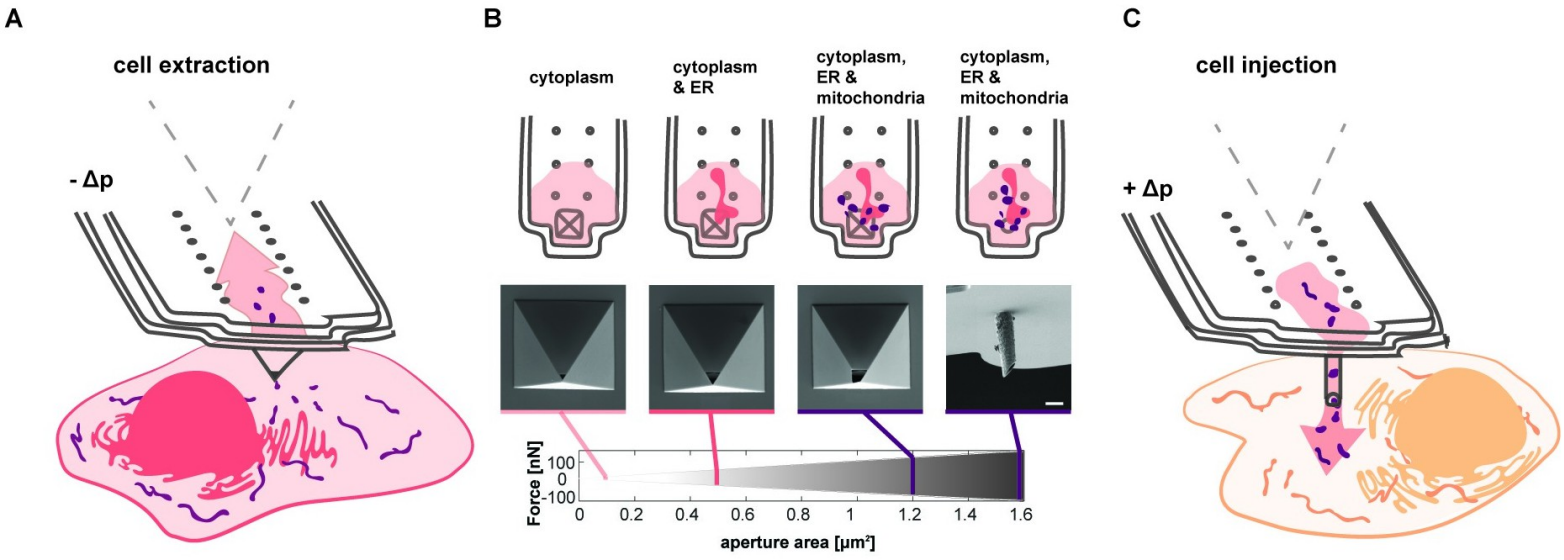
Schematic of organelle extraction and injection using FluidFM. (**A**) Extraction volumes are tuned by applying negative pressure (−Δp). Probe prefilling with octadecafluorooctane allows for optical and physical separation of the extract within the cantilever. (**B**) Selective extraction of organelle components by tuning the aperture size and thus the applicable range of applied fluidic forces. Top row: schematic view of extracted cell components inside the cantilever. Middle row: scanning electron microscopy images of cantilever apexes with different apertures. Bottom row: range of applicable fluidic forces with adapted FluidFM cantilevers. Scale bar: 2 μm. (**C**) Schematic of mitochondria injection into single cells by applying positive pressure (+Δp) once the cantilever was inserted into the recipient cell. The data underlying Fig 1B can be found in S1 Data. ER, endoplasmic reticulum.

**Fig 2:**
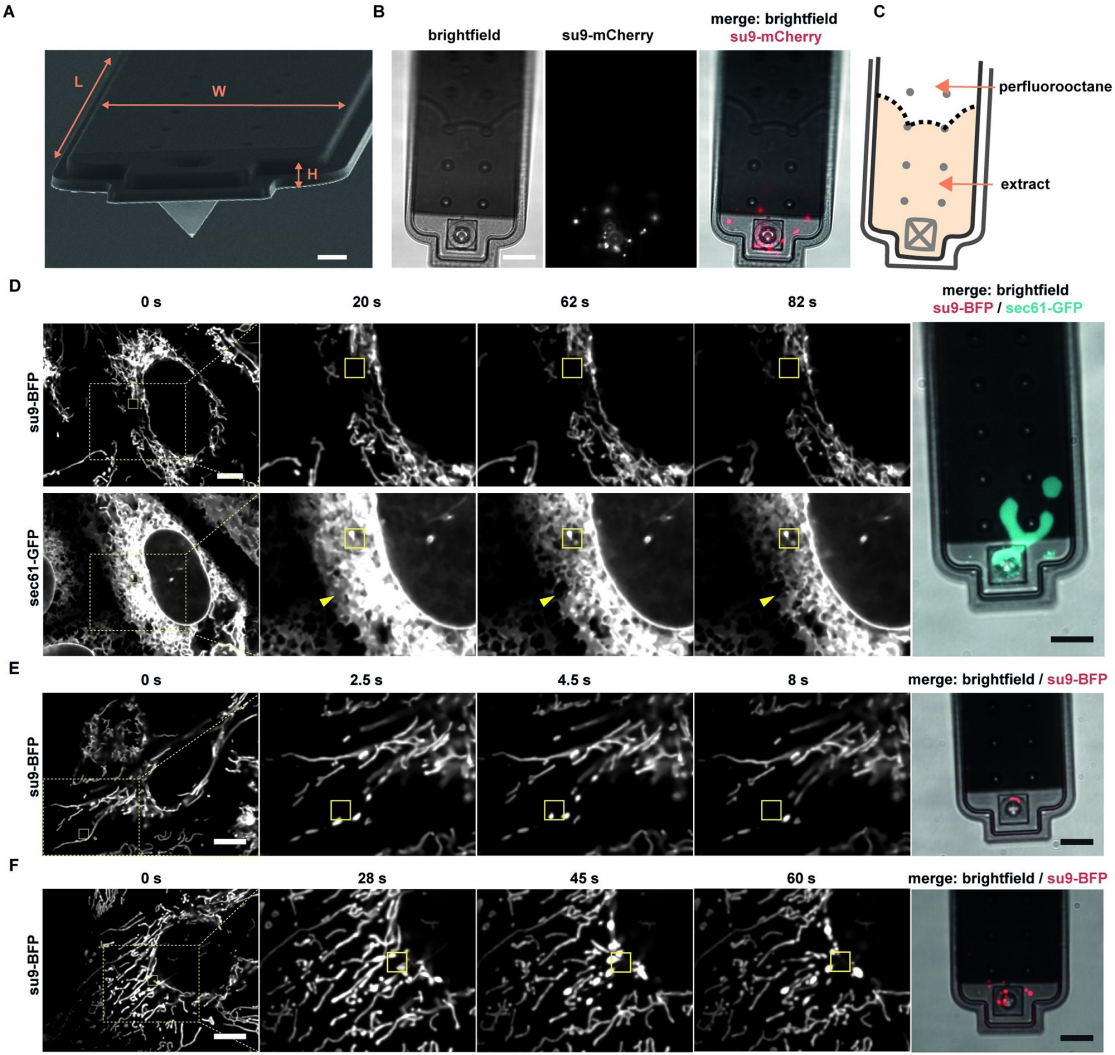
Organelle extraction. (**A**) Scanning electron microscopy image of a FluidFM cantilever. Channel dimensions: L = 200 μm, W = 35 μm, H = 1 μm. Scale bar: 5 μm. (**B**) Fluorescence microscopy images of a FluidFM cantilever after mitochondria extraction. The border between the extract and perfluorooctane can be seen due to different refractive indexes. Scale bar: 10 μm. See also S1 Movie. (**C**) Scheme of the extract and perfluorooctane inside the cantilever shown in (B). The extract occupies a volume of 1170 fL. (**D**-**F**) Time-lapse images of live cell compartment extractions and z-stacks of pyramidal cantilevers post-extraction. Yellow boxes indicate position of the cantilever apex inside the cell. (D) U2OS cell expressing su9-BFP (mitochondria) and Sec61-GFP (ER, cyan) during extraction. Arrows indicate the zone of ER retraction. A cantilever with an aperture of 0.5 μm^2^ was used. Scale bar: 10 μm. See also S1 and S2 Movies. (E) Extraction of a single mitochondrion from a viable U2OS cell expressing su9-BFP. A cantilever with a 1 μm^2^ aperture was used. Scale bar: 10 μm. See also S3 Movie. (F) Extraction of several mitochondria from a viable U2OS cell expressing su9-BFP. A cantilever with a 1 μm^2^ aperture was used. Scale bar: 10 μm. See also S4 Movie. ER, endoplasmic reticulum.

Here, we overcome these limitations of organelle manipulation. To do so, we have established FluidFM [23] as a single-cell technology for intra- and intercellular micromanipulation of organelles in living cells (Fig 1A–1C). FluidFM combines the high-precision force-regulated approach of an atomic force microscope (AFM, pN to μN) with the volumetric dispensing of nanoscale pipets (fL to pL) under optical inspection, providing the forces and volume control relevant for single-cell manipulation [24]. These features are unique among miniaturized probes and pivotal for driving the probe into the cytosolic compartment in a minimally invasive manner. Organelle extraction and injection achieved here required dedicated fabrication of tips with customized aperture area (*A*), to both overcome steric constraints and increase the range of applicable suction forces (*F*_*max*_) at the aperture via the fluidic pressure (Δ*p*) in accordance to *F*_*max*_ = Δ*p* × *A*. We adapted the size and geometry of the aperture and microchannel of the FluidFM probes and used the tips in combination with force control for membrane insertion via automated AFM and exertion of suction (i.e., tensile) forces via automated pressure controller. We simultaneously inspected target cells and the transplant inside the transparent cantilever (via phase contrast and fluorescence microscopy, Fig 2A–2C). We demonstrate the establishment of a robust and maximally efficient method for the extraction of mitochondria from living cells and for functional transplantation between individual cells. The gentle mode of probe insertion and extraction ensures cell viability and allows monitoring of mitochondrial dynamics in real time and across cell generations and tracking mitochondrial variants and fate in recipient cultured and primary cells.

## Results

### Tunable organelle extraction from live cells

To enable organelle manipulations, in particular their unconstrained flow through the probe, we manufactured FluidFM probes with a channel height of 1.7 μm and drilled apertures up to 1,100 nm × 1,100 nm (*A* = 1.2 μm^2^) with focused ion beam (FIB) compared to the diameter of mitochondria of 300 to 800 nm [25]) (Fig 1B). In addition, we designed and fabricated dedicated probes with a cylindrical tip to facilitate minimally invasive cell entry. These were sharpened by FIB milling to resemble a hollow needle to facilitate membrane insertion (Fig 1B, S1 Fig). These probes had an aperture area of *A* = 1.6 μm^2^, further minimizing steric limitations and increasing the range of applicable hydrodynamic forces spanning from a few pN to over 100 nN, while showing great robustness toward mechanical stress (S2 Fig). The general workflow for the manipulation of intracellular membrane enclosed compartments involves positioning the FluidFM probe above a selected subcellular location and their insertion by AFM force spectroscopy, followed by either extraction of material from the cell by exerting negative pressure (Fig 1A, S1 Movie) or injection into the cell by positive pressure (Fig 1C). Exclusion of large organelles is achieved by fine-tuning the aperture size (*A*) and the strength of the applied negative pressure (−Δ*p*). The extraction of cytoplasmic material is monitored in real time, and the extract is inspected inside the FluidFM channel by optical microscopy after relieving the pressure (Δ*p* = 0) and retracting the probe (Fig 2A–2C, S1 Movie). Force feedback by the AFM enables flexible extraction and injection times (seconds to minutes) during which the probe can be maintained inside the cell cytoplasm without impairing cell viability, allowing extraction and delivery of solutions that represent large fraction of the manipulated cells’ own volume (up to 90%) [26]. FluidFM probes with a channel height of 1 μm can capture a total volume of 7 pL. The extraction process allows exclusion of organelle compartments that require larger apertures and higher hydrodynamic forces for extraction. However, when larger apertures are used, smaller and less strongly crosslinked organelles will be coextracted (Fig 1B).

The sampled material can be dispensed subsequently for downstream analyses or transplanted directly into a recipient cell (Fig 1). To examine the capabilities of the newly fabricated FluidFM probes for organelle sampling from single cells, we monitored the endoplasmic reticulum (ER) and mitochondria. We used human osteosarcoma epithelial (U2OS) cells and visualized in parallel the ER by expression of GFP fused to the resident protein Sec61β (sec61-GFP) and mitochondria by expression of BFP targeted to the mitochondrial matrix (su9-BFP). When utilizing pyramidal probes with an aperture size of *A* = 0.5 μm^2^ and low pressure offsets, Δ*p* < 20 mbar, we accumulated ER in the cantilever, which was accompanied by disappearance of GFP signal in the cell (Fig 2D, S2 Movie). During extraction, the ER was pulled toward the cantilever tip, and we observed a general conversion of cisternal to tubular ER, in both U2OS cells and a similarly labeled kidney cell line (COS7, S3 Fig, S2 and S3 Movies). Notably, under these conditions, the mitochondrial network remained unperturbed, and mitochondria were not extracted.

Next, we aimed at extracting mitochondria using pyramidal probes with a larger aperture size (*A* = 1.2 μm^2^) and newly developed, slanted cylindrical probes (*A* = 1.6 μm^2^) (Fig 1B). Tunable extraction of mitochondria was achieved using both kinds of probes, thus enabling aspiration of individual mitochondria or sampling of larger quantities of the mitochondrial network (Fig 2E and 2F, S4 and S5 Movies).

We examined cell viability upon subcellular manipulation of ER and mitochondria and did not find it compromised (>95% cell viability) (S4 Fig). To further ensure that our extraction protocol does not damage the cytoplasmic membrane upon probe insertion, we conducted a dedicated set of experiments and monitored potentially occurring Ca^2+^ influx from the cell culture medium using a fluorescent probe (mito-R-GECO1 [27]). Our experiments confirmed that there was no ion influx during and after manipulation, indicating integrity of the cytoplasmic membrane during organelle extraction (S7 and S8 Movies).

Monitoring mitochondrial extraction, we noticed that mitochondrial tubules exposed to tensile forces (negative pressure) underwent a shape transition reminiscent of a “pearls-on-a-string phenotype” [28] inside the cytoplasm of the target cells. This phenotype was characterized by discrete spheres of mitochondrial matrix, connected by thin and elongated membrane stretches (S5A and S5B Fig). These globular structures eventually pinched off upon further exertion of a pulling force and resulted in spherical shaped mitochondria in the cantilever (Fig 2E, S5A–S5C Fig). To date, it was not possible to exert hydrodynamic forces intracellularly, while distinguishing physical manipulation from other potential cellular triggers. The observed mitochondrial “pearls-on-a-string” phenotype was previously described to result from calcium overflow [28] or mitochondrial membrane rupture [29]. To ensure mitochondrial membrane integrity and thus functionality, we investigated whether both mitochondrial membranes remained intact during the process. To this aim, we used U2OS cells and performed time-lapse microscopy during extraction of mitochondria in which both the matrix (su9-BFP) and the outer mitochondrial membrane (OMM; Fis1TM-mCherry) were labeled. Consistent with the observations described above (Fig 2E), we observed the morphological change of mitochondrial tubules exclusively under direct fluid flow at the cantilever aperture and thus exertion of tensile forces, but not upon probe insertion without fluid flow (S5A and S5B Fig, S5 and S6 Movies). Our data further show that the force-induced shape transition propagated over tens of micrometers along the mitochondrial tubules in the millisecond to second range after negative pressure was applied with FluidFM (S5B and S5C Fig, S6 Movie). The shape transition of the matrix compartment propagated homogeneously along connected mitochondrial tubules, while the OMM between the matrix foci initially remained intact. When traction was maintained for a few seconds, the OMM separated at one or more constriction sites between previously formed “pearls”, which resulted in isolated spherical mitochondria, while the remainder of the tubular structure relaxed and recovered (S5A–S5C Fig, S6 Movie). Furthermore, we demonstrate that the observed scission process of “pearling” mitochondria (S5C Fig) succeeds recruitment of the fission machinery protein GTPase dynamin-related protein 1 (Drp1) [30] to the constricted sites (S1 Text, S5D Fig). Combining mitochondrial pulling experiments with mitochondria-localized calcium sensors, we were able to show that the shape transition toward the pearls-on-a-string phenotype and subsequent mitochondrial fission inside the cytoplasm was calcium-independent (S6 Fig, S7–S10 Movies) (S1 Text, S5 and S6 Figs, S7 and S8 Movies).

### Mitochondrial transplantation into cultured cells

**Fig 3:**
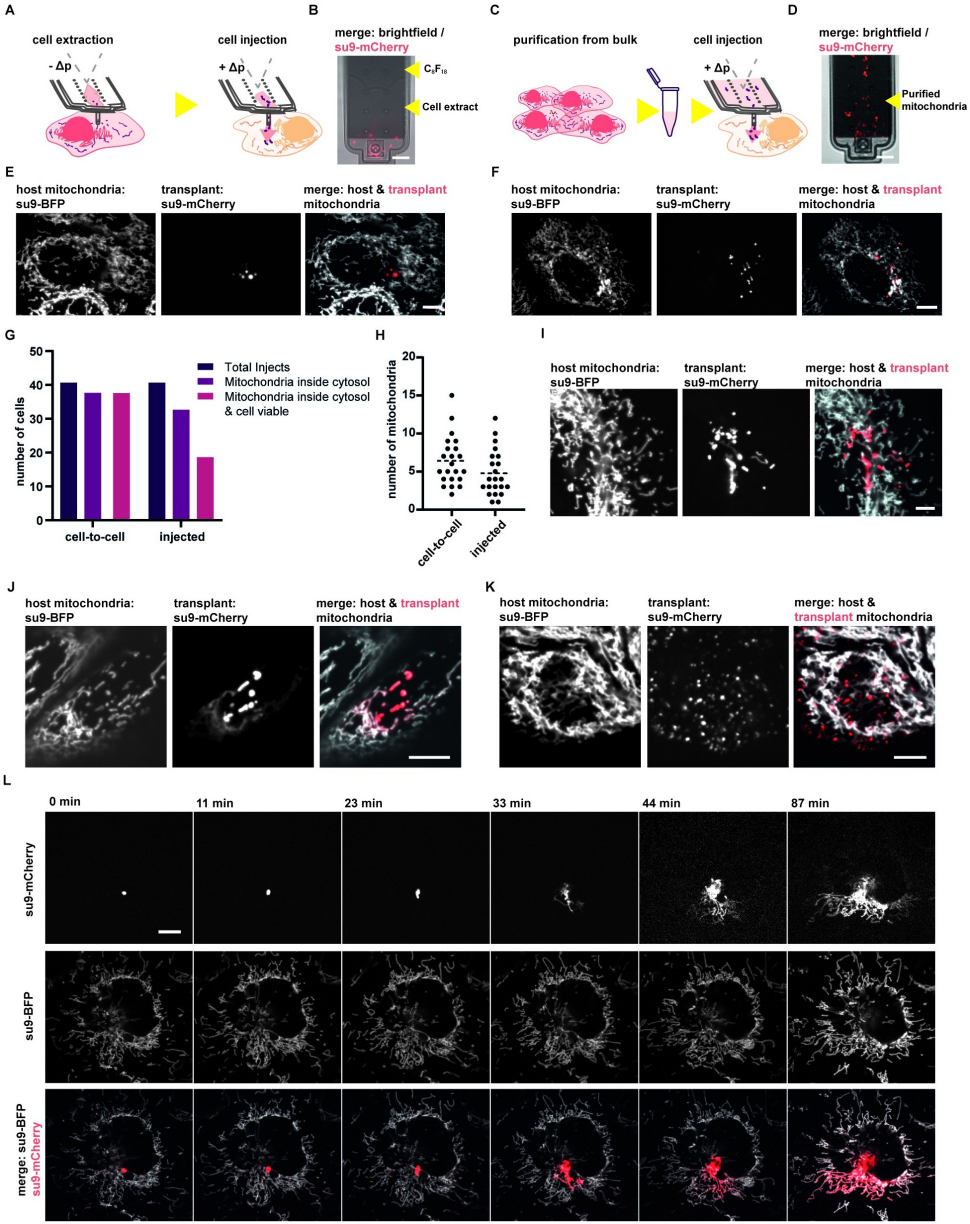
Mitochondrial transplantation. (**A**) Scheme of mitochondrial transplantation using the cell-to-cell transfer approach: Mitochondria are extracted via FluidFM aspiration. Subsequently, the cantilever holding the extract is moved to a recipient cell and the extract is injected. (**B**) Image of a FluidFM cantilever prefilled with perfluorooctane after mitochondrial extraction, mitochondria are labeled via su9-mCherry. Extracted volume approximately 0.8 pL. Scale bar: 10 μm. (**C**) Scheme of mitochondrial transplantation using mitochondria purified by a standard mitochondrial purification protocol. The purified mitochondria are resuspended in HEPES-2 buffer and filled directly into the fluidic probe. Cells are injected consecutively. (**D**) Image of a FluidFM cantilever filled with mitochondria isolated from bulk, labeled via su9-mCherry. Scale bar: 10 μm. (**E**) Images of a recipient cell post–mitochondrial transplantation via the cell-to-cell approach. The host cells’ mitochondrial network is labeled via su9-BFP, and the transplant is labeled via su9-mCherry. Scale bar: 10 μm. (**F**) Images of a recipient cell post–mitochondrial transplantation via the injection of isolated mitochondria approach, labels similar to c. Scale bar: 10 μm. (**G**) Evaluation of mitochondrial transplantation via the cell-to-cell approach upon optical inspection and the injection of isolated mitochondria approach. A total of 40 cells were evaluated per approach. (**H**) Absolute numbers of transplanted mitochondria of 22 individual cells evaluated for the cell to cell and the injection of isolated mitochondria approach. (**I**) Fusion states of transplanted mitochondria 30 post–cell-to-cell transplantation. Mitochondria are visualized using different fluorescent labels for the transplant (su9-mCherry) and for the host mitochondrial network (su9-BFP). Scale bar: 5 μm. (**J**) Fusion states of transplanted mitochondria 30 postinjection of purified mitochondria, similar labellng as in g. Scale bar: 5 μm. (**K**) Degradation of transplanted mitochondria, the transplant is split into multiple smaller fluorescent vesicles (su9-mCherry) showing no overlap of fluorescence with the labeled host cell mitochondrial network (su9-BFP). Scale bar: 5 μm. (**L**) Time-lapse image series of a single transplanted mitochondrion (su9-mCherry). The organelle donor was a HeLa cell, recipient cell is a U2OS-cell with a fluorescently labeled mitochondrial network (su9-BFP). Scale bar: 10 μm. The data underlying Fig 3G and 3H can be found in S1 Data.

Our next goal was to demonstrate the functional delivery of mitochondria into new host cells and to achieve cell-to-cell organelle transplantation. In contrast to mitochondria extraction, for which both pyramidal probes and cylindrical probes could be used (Fig 1B), injection of mitochondria was possible only with the latter, newly developed probes. FluidFM offers 2 possibilities for mitochondrial transfer: transplanting mitochondria from a donor cell to a recipient cell by coupling mitochondrial extraction with reinjection of the extract into a new host cell or back-filling FluidFM probes with mitochondria purified by subcellular fractionation, followed by injection (Fig 3A–3D). Working with bulk-isolated mitochondria allows for a higher throughput of cells injected in series with one cantilever (>1 cell per minute). However, such a protocol is accompanied by reduced mitochondrial quality caused by the preceding purification process. We compared both approaches, the cell-to-cell approach (Fig 3A) and the injection of purified mitochondria (Fig 3C), with respect to the delivery of mitochondria into the cytoplasm of individual cultured HeLa cells.

To visualize the transfer of mitochondria, we used donor and acceptor cells with a differentially labeled mitochondrial matrix (Fig 3E and 3F; su9-mCherry and su9-BFP, respectively). When transplanting mitochondria directly from cell to cell using FluidFM, we achieved successful transfer of mitochondria into the cytosol of the recipient cells in 95% of all cases, while maintaining cell viability (Fig 3G, 39 out of 41 transplanted cells). Upon injection of purified mitochondria, we observed mitochondrial transfer and preserved cell viability in 46% of cases (19 of 41) (Fig 3G, S7 Fig). Quantification of the transplant showed that the number of transplanted mitochondria for these experiments varied from 3 to 15 mitochondria per cell (Fig 3H). The different success rates between the 2 alternative protocols can be explained by differences in mitochondrial condition. When evaluating mitochondrial extraction protocols, we observed that a fraction of the extracted mitochondria undergo rupture of their outer membranes (S7 Fig) [31]. Irreversible damage of mitochondria leads to degradation inside cells and potentially cell apoptosis due to cytochrome c leakage. While cell-to-cell transplantation of mitochondria reduces throughput, it has the advantage that the extracellular time is short (<1 minute) and that mitochondria sampled by FluidFM are maximally concentrated in native cytoplasmic fluid, bypassing the use of artificial buffers altogether. We ensured that the extract remained near the aperture during extraction by filling the probes with immiscible perfluorooctane before extraction and transplantation. Therefore, only small volumes (0.5 to 2 pL) are injected into the host cells (Fig 3B), up to the volume previously extracted from the donor cell (injection of larger volumes is automatically prevented due to inherent flow resistance properties of the prefilled fluorocarbon liquid).

Labeling mitochondria of the recipient cell (su9-BFP) in addition to labeling donor cell mitochondria (su9-mCherry) allowed us to survey the state of the mitochondrial network in the transplanted cell. In both transfer approaches described above (transplantation and purification followed by injection), the tubular, interconnected phenotype of the host mitochondrial network remained unaltered by the injection process. In addition, labeling allowed us to monitor the fate of the transplanted mitochondria. We observed mitochondrial acceptance, defined by fusion of the transplant with the host mitochondria network, and thus overlap of both fluorescent signals and mitochondrial degradation, marked by further fragmentation of the transplant and segregation into presumptive mitophagosomal structures (Fig 3I–3K). These processes were observed irrespective of the transfer method, cell to cell (Fig 3I), or injection of purified mitochondria (Fig 3J). We followed the fate of the transplant over time in 22 cells: 18 cells showed full mitochondrial fusion of the transplant, and 4 cells showed mitochondrial degradation. Fusion events were first observed within 30 minutes posttransplantation in most cases (14 of 18 cells).

As indicated above, the high cell-to-cell transplant efficiency allows for direct observation of the fate of individually transplanted mitochondria. To showcase this, we transplanted labeled mitochondria (su9-mCherry) from HeLa cells into differentially labeled U2OS cells (su9-BFP), regularly used for studies of dynamic mitochondrial behavior. The strong label together with a highly sensitive microscope camera enables tracing of individual mitochondria within a recipient cell over time (Fig 3I, S11 Movie). In this case, we observed rapid spread of the fluorescent mitochondrial matrix label after the initial fusion event to the network 23 minutes posttransplantation.

In summary, we established 2 methods for mitochondria transfer into single cultured cells. One involves bulk purification of mitochondria and their injection into recipient cells. The injection protocol is rather rapid but inevitably compromises mitochondrial and cellular function. The second consists in cell-to-cell transplantation. Evaluation of cell viability and transplant fate showed an efficient protocol that allows observation of the dynamic behavior of transplanted mitochondria after transfer.

### Fate of transplanted mitochondria in primary cells

Having developed an efficient protocol for cell-to-cell transplantation of mitochondria, we sought to test whether primary cells show similar uptake behavior as the tested cancer cells and, if so, what are the dynamics of integration of foreign mitochondria. We considered these particular experiments important because quality control mechanisms are impaired in cancer cell lines [32] and to demonstrate the broad applicability of the established protocol. In addition, several studies link naturally occurring mitochondrial transfer events with short-term benefits for individual cells and tissues, for example in osteocytes [33], adipose tissue [34], or in neurons [35]. However, to the best of our knowledge, the fate of mitochondria or dose-response relationships have not been studied, and appropriate technologies of mitochondrial transfer that preserve cell viability have been lacking.

**Fig 4:**
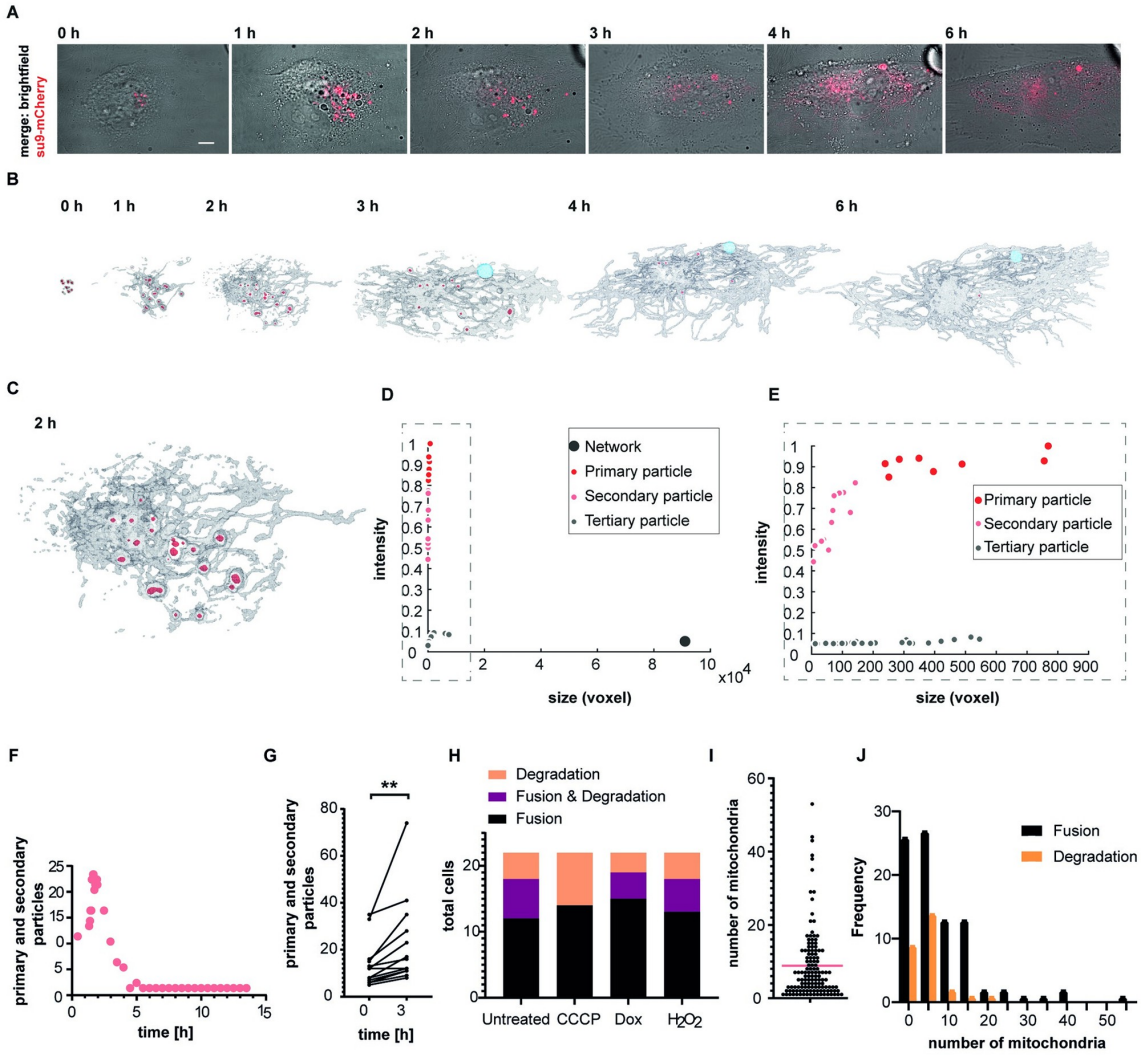
Mitochondrial fate in HEKa cells. (**A**) Time series of cell-to-cell transplantation from HeLa (su9-mCherry) to HEKa, fusion and degradation to the unlabeled host cell network. Scale bar: 10 μm. (**B**) Surface-render (total su9-mCherry signal) of the time-lapse series of mitochondrial fusion and degradation within the host cell shown in a. The transplant is depicted in red, host cell mitochondrial network carrying fluorescent traces of the transplant is shown in gray, presumptive mitophagosomal structure is shown in light blue. (**C**) Enlarged section of the 2 hours time point surface render from b. (**D**) Scatter plot of size and normalized fluorescence intensity distribution of objects detected at the 2-hour time point. (**E**) Zoom in on low-size fraction of the scatter plot shown in c. (**F**) Total number of objects classified as primary transplant or secondary particle over time. (**G**) Absolute number of primary and secondary particles detected directly after transplantation (0 hour) and after 3 hours for 12 cells. Paired *t* test, *p* = 0.0095. (**H**) Fusion and degradation behavior of drug-compromised mitochondrial transplants in HEKa cells. Each condition was tested with 22 cells. (**I**) Distribution of mitochondrial quantity transplanted per cell from all conditions tested in e, total number of cells: 132. Dashed line indicates mean value. Total number of transplanted mitochondria: 1,117. (**J**) Histogram plot correlating the amount of transplanted mitochondria with uptake outcome; bin width is 5. Correlation of the absolute number of transplanted mitochondria per cell with the cell response of either mitochondrial fusion or degradation across all transplant conditions in percent. Total cell number: *n* = 135. The data underlying Fig 4D–4J can be found in S1 Data. HEKa, human endothelial keratinocyte.

We used primary human endothelial keratinocytes (HEKa), a skin cell type that is generally susceptible to radiation damage and aging [36]. In standard culture conditions, the mitochondrial network of HEKa cells is mostly tubular, forming a large connected network (S8 Fig) similar to HeLa cells studied above, indicating an active mitochondrial fusion machinery [37]. Notably, cell-to-cell transplantation of mitochondria into HEKa cells showed no impact on their viability, allowing analysis of all injected cells. We conducted time-lapse experiments of transplanted labeled mitochondria (su9-mCherry) from HeLa cells into HEKa cells, focusing on the fluorescent signal of the transplant and its dynamic behavior over time (Fig 4A–4C, S12 Movie).

The analysis revealed the rate of transplant fusion with the host mitochondrial network and its movement inside the cytosol. In contrast to the findings described above for HeLa-to-HeLa transplantations, i.e., host cells either accepting or degrading mitochondria (S9 Fig), we observed a third, intermediate scenario in which single cells both fused and degraded parts of the transplant. To further define the transplant uptake mechanisms in HEKa cells, we classified the transplanted mitochondria as primary particles—mitochondria that retain their original shape and fluorescence intensity, indicating that they have neither fused with the host network, nor been targeted to degradation—secondary particles—mitochondria that retain their fluorescence but are distinctively smaller in size, suggesting fragmentation for degradation—and tertiary particles—mitochondria fused with the host network, showing a characteristic decrease of transplant specific fluorescence (>10-fold), due to dilution into the host mitochondrial network (Fig 4D and 4E, S12 Movie). Shape transition of the transplant from discrete spheres toward tubules was observed exclusively in the context of fusion of the transplant to the existing host network. Emergence of secondary particles hinted toward rearrangement of the transplant into parts recognized as “viable” mitochondria, designated for fusion to the network, while another part was targeted for degradation. Tracing this process within an individual cell, we counted the number of “primary” and “secondary” particles over 14 hours (Fig 4F). In the first 3 hours postinjection, we observed an increase in “primary” and “secondary particles”, which subsequently lost their high fluorescent signature while the fraction of fluorescent mitochondrial network increased over time (Fig 4B–4E). To test whether restructuring of the transplant into subparticles was common in cells that showed emergence of secondary particles, we counted the number of primary and secondary particles immediately after transplantation and 3 hours after in cells that showed both degradation and integration of the transplant (Fig 4G; *n* = 10). The results indicated variance in the absolute extent of rearrangement; however, all cells showed an increased particle number by a factor of 1.7 ± 0.4.

In this first set of transplantation experiments from HeLa cells to HEKa cells, 12 cells showed complete uptake of the transplant, 6 showed both uptake and degradation of the transplant, and 4 fully degraded the transplant (Fig 4H). The degradation of defined amounts of transplanted mitochondria can be traced over time in single cells, as exemplarily shown in S10 Fig.

In the experiments described above, the first fusion or degradation events occurred 20 minutes posttransplantation and continued for more than 16 hours. Remarkably, the speed at which the primary transplant was processed was independent of transplant sizes. Fusion or degradation of large transplants, >40 mitochondria per cell, advanced at similar rate as smaller transplants, <8 mitochondria per cell (S12 Movie, S11 Fig).

As outlined above, the established cell-to-cell transplantation protocol is minimally invasive regarding the integrity of the mitochondria themselves when using “healthy” donor cells, and cells receiving transplants from unperturbed donor cells showed mostly uptake of the transplant (Fig 4H). Next, we wondered whether the acceptance of mitochondria by host cells was altered, if the quality of the transplant was impaired by prior drug treatment of the donor cell. Such treatments are commonly used to study the pathways that control the maintenance of the mitochondrial network. However, drugs have been applied to the entire cell so far, likely interfering with regulatory processes of cells as a whole. In consequence, the here established cell-to-cell transplantation of mitochondria provided an opportunity to follow the fate of damaged mitochondrial subpopulations in the context of an otherwise intact cell. In vitro, the chemical triggers used to study mitophagy are uncoupling agents, inhibitors of the respiratory chain or combinations thereof, robustly causing changes of membrane potential and simulating low mitochondrial quality [38]. To investigate how otherwise healthy cells respond to impaired mitochondrial subpopulations, we treated transplant donor cells with the proton ionophore CCCP (S8 Fig) and transplanted the depolarized mitochondria into HEKa cells. Most cells, 14 of 22, showed fusion of initially depolarized mitochondria to the network, while 8 cells degraded the transplant (Fig 4H). This reaction was similar to the previous condition tested, the transplantation of untreated mitochondria, which was unexpected, because literature suggests rapid mitochondrial degradation of mitochondria having lost their membrane potential [39,40]. However, the membrane potential can potentially recover quickly, if a functional ATPase is present, as expected in our study. Next, we tested the influence of doxycycline, an antibiotic, inhibiting protein synthesis of the mitochondrial ribosome, which induces fractionation of the mitochondrial network in HeLa cells after 24 hours (S8 Fig), putatively due to nonfunctional mitochondrial OXPHOS protein complexes [41]. The reaction of the host cells was similar as toward depolarized and untreated mitochondria: 15 show full fusion, 4 show both fusion and degradation, and 3 show full degradation of the transplant. We then treated donor cells with H_2_O_2_, which damages proteins and induces double strand breaks in mtDNA. The fraction of mtDNA containing double strand breaks in H_2_O_2_ treated cells was reported to remain high for several hours posttreatment [42], and the donor cells showed a fragmented mitochondrial network after 3 hours (S8 Fig). However, even upon H_2_O_2_ treatment, and even upon application of all drugs simultaneously to donor cells, mitochondrial acceptance in healthy recipient background was still comparable to all other conditions, indicating the potential of cells to cope with highly damaged mitochondria when occurring as isolated events (Fig 4H, S12 Fig).

Since the tested conditions for drug-impaired mitochondria did not show an impact on mitochondrial uptake behavior, we wondered whether the amount of injected mitochondria might have an impact on transplantation outcome. Across all conditions, we transplanted 1,117 mitochondria (Fig 4I) and followed their fate in more than 100 individual primary cells after transplantation of 1 up to 53 mitochondria (S12 Fig). Pooling uptake data of cross-tested conditions (*n* = 135 cells), we plotted the frequency of cells showing transplant fusion and transplant degradation versus the number of individually transplanted mitochondria and could not find any correlation between the number of transferred mitochondria per cell and their uptake behavior (Fig 4J).

Overall, transplantations were successful in all experiments and cells remained viable, irrespective of the amount of transplant received. We demonstrated that primary HEKa cells incorporate a majority of transplanted mitochondria into their network via mitochondrial fusion. A subset of transplanted cells showed individualized responses, with one fraction of mitochondria fusing with the mitochondrial network and another subpopulation undergoing rearrangement associated with particle formation, while a third fraction was subjected to degradation. This behavior highlights the presence of quality control mechanisms that are in place to sort each mitochondrion and exemplifies the potential of the FluidFM approach to transplant mitochondria into new host cells to study their fate and host cell response with single mitochondria resolution.

### Mitochondria transplantation and transfer of mitochondrial genomes

**Fig 5:**
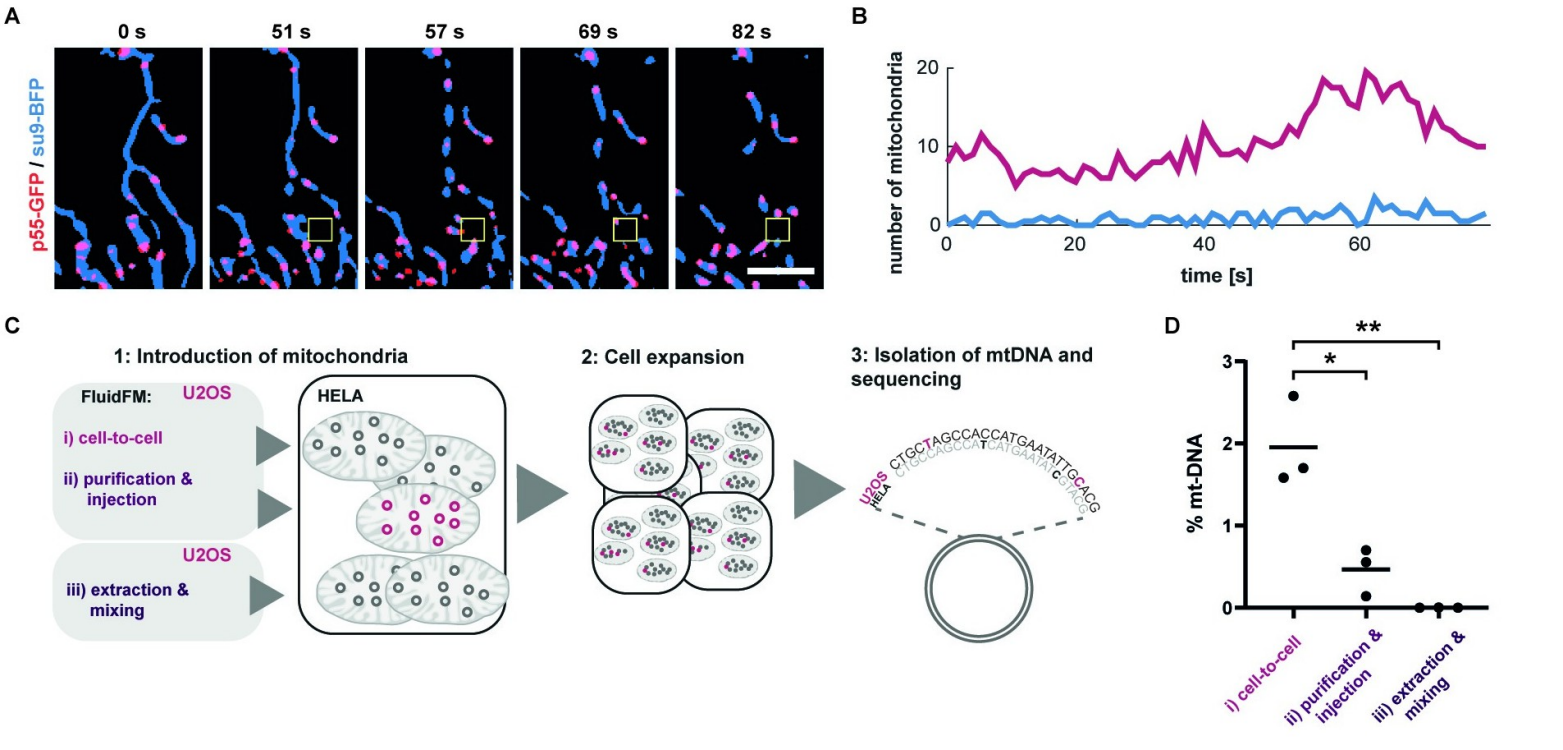
Mitochondria transplantation and transfer of mitochondrial genomes. (**A**) Time-lapse evaluation of mitochondria extraction visualizing mitochondrial nucleoids. An overlay of mitochondrial matrix shown in blue (su9-BFP) and mitochondrial nucleoids shown in red (p55-GFP). Yellow box indicates position of the cantilever. **(B**) Dynamic localization of labeled nucleoids within a fractionating mitochondrial network. Plotted are the total amounts of mitochondria (su9-BFP) that show overlap with a labeled nucleoid (p55-GFP) in red and idle mitochondria showing no fluorescent trace of p55-GFP in blue over time. See also S13 Fig. Scale bar: 5 μm. (**C**) Strategy for the quantification of mtDNA maintenance after mitochondrial transplantation. (**D**) Quantification of retained U2OS mtDNA in transplanted HeLa cells of all approaches tested. Bars indicate mean value. Difference between condition i and ii, *p* = 0.0138 and between condition i and iii, *p* = 0.0034. The data underlying Fig 5B and 5D can be found in S1 Data. mtDNA, mitochondrial DNA.

After demonstrating the short-term response of cells to mitochondrial transplantation, we focused on the long-term effects of mitochondrial transfer over generations of host cells. Mitochondria differ from other membrane compartments in that they carry their own genome, which is propagated within the cell’s inherent mitochondrial pool. It has been shown that mtDNA can be transferred into somatic cells via miniaturized probes under selective pressure, either by transfer into cells artificially rendered free of mitochondrial DNA (rho zero cells) [19,22] (2% to 25% efficiency) or by selection using antibiotics (<0.01% efficiency) [9]. Therefore, the introduction of new mtDNA sequences into functional somatic cells remains a challenge [20,43]. Before demonstrating the transfer of mtDNA into host cells upon transplantation, we first wondered whether the FluidFM-extracted mitochondria contain mitochondrial DNA, which is organized in discrete complexes termed mitochondrial nucleoids, because the extraction process leads to rapid fragmentation of the network. To visualize the behavior of mitochondrial nucleoids during mitochondrial extraction using FluidFM, we expressed a fluorescently tagged version of p55 (p55-GFP), a polymerase-γ subunit that colocalizes with mitochondrial nucleoids and appears in discrete speckles scattered throughout the mitochondrial matrix [44] (Fig 5A).

We evaluated time-lapse experiments upon mitochondrial extraction with labeled nucleoids and counted mitochondrial fragments either containing labeled nucleoids or being devoid of their fluorescent signal (Fig 5B, S13 Fig). We observed p55-GFP in >90% of formed mitochondrial spheres (*n* = 18), indicating that most transplanted mitochondria contained mtDNA.

Next, we applied genomics to quantify uptake and maintenance of mtDNA after mitochondria transplantation (Fig 5C). We compared 2 methods of mitochondrial transfer, using U2OS cells as mitochondria donors and HeLa cells as acceptors: direct cell-to-cell transplantation of mitochondria with FluidFM and FluidFM injection of purified mitochondria. In addition, to control for nonspecific uptake of mitochondria or otherwise unspecific mtDNA carryover, we seeded cells into a culture dish and mixed them with extracted mitochondria from donor cells. All experiments were executed in biological triplicates and cells were passaged onto a fresh culture dish; for a more detailed description of the experimental procedure and conditions, see Methods section. Subsequently, we extracted DNA, then amplified and sequenced part of the variable D-loop region of the mitochondrial genome. Four single nucleotide polymorphisms [45] allowed the detection of transplanted mtDNA. The percentage of transplant mtDNA propagation was highest in samples from cell-to-cell mitochondria transplantation (mean 2%), followed by FluidFM injection of purified mitochondria (mean 0.5%), whereas mtDNA transfer by nonspecific uptake was below the detection limit (Fig 5D, S1 Table). The obtained amount of mtDNA was well in line with our expectations considering the amount of mtDNA copies per cell (approximately 1,100) [46] and mtDNA copy number per nucleoid (1.4 to 3.2) [47,48], and the quantity of mitochondria we transplanted within this experimental series (Fig 3H, median = 6).

In summary, we showed efficient transplantation of mitochondrial DNA into somatic cells without the need for selective pressure. These results indicate that HeLa cells show little or no selection for mitochondrial DNA variants of another cell line, here U2OS cells, under standard culture conditions.

## Discussion

The technology developed here allows the manipulation of intracellular compartments using FluidFM. We show the removal and injection of organelles from and into single cells without compromising organelle integrity nor cell viability. The scalability of the volume of the subcellular sampling protocol for organelles allows molecular downstream analyses, which are increasingly feasible thanks to the improving sensitivity of “omics” technologies [49]. Beyond the showcased manipulation of ER and mitochondria, the technology in principle allows sampling and introduction of other cellular structures like the Golgi apparatus, stress granules, or protein aggregates. Together with sensitive omics approaches [50,51], extraction of fractions of local organelle structures may enable analysis of organelle heterogeneity within single cells. Homeostatic cell states such as cell size or organelle content can be studied by manipulating individual cells at one or multiple time points. We demonstrate the application of FluidFM to exert localized fluidic forces within single cells and thus expand the possibilities for subcellular sampling, as well as the study of organelle mechanobiology [16,26]. It has been proposed that mechanical forces and membrane constriction affect mitochondrial shape and dynamics [52,53], but with the previously established tools, it has been difficult to test such a hypothesis without perturbing the cellular state as a whole [29,52]. Gonzalez-Rodriguez and colleagues [29] suggest that the shape of mitochondria depends on the elastocapillary number (*N_ec_*), which describes the ratio between the elastic modulus (*E*) and mitochondrial membrane tension (*γ*): Nec≡E×dγ; where d is the membrane thickness. A decreasing *N*_*ec*_ results in “pearling” [29,54]. This description explains our observations that pulling on the mitochondrial membrane (S5A Fig) increases the mitochondrial membrane tension, thus decreasing *N*_*ec*_. While the application of compressive force can be controlled in time and space [55], application of controlled tensile force has been impossible to date. Here, we demonstrate that mechanical force can be a driver for mitochondrial shape transition that is strictly localized to sites of tensile force application and propagates along membrane-connected mitochondrial tubes. “Pearling” eventually leads to recruitment of Drp1 and mitochondrial fission. In a more physiological setting, the transition into the pearls-on-a-string phenotype appears to be an elegant solution that protects cells against membrane leakage during mechanical stress.

The purely mechanical nature of FluidFM presents itself as a strength, allowing dissection from complex physiological stimuli, often involving calcium-influx, from isolated exertion of (hydrodynamic) pulling forces. The new cylindrical FluidFM probes will also be a powerful tool to study cellular mechanoreception, a field that has recently gained attraction due to the creation of novel membrane tension sensors [56].

Transplantation of mitochondria at a high efficiency allowed us to track organelle fate over time in new genetic and physiological cell backgrounds. Similar to organ transplants that are accepted or rejected by new hosts, here, we show rescue or failure of organelles within single cells after transplantation. We show that transplantation is highly efficient (95% success rate) when mitochondria are transplanted directly between living cells rather than using isolation protocols prior to transfer.

The FluidFM-based approach of efficient mitochondria transplantation permitted us to evaluate mitochondrial quality control in primary cells by transplanting healthy and compromised mitochondria and observing their fate. In addition, we show that individual cells generally differentiate between individual mitochondria, but do not display apparent responses to mitochondria previously exposed to the tested stresses. The heterogeneous response toward the injected mitochondrial subpopulation might hint toward a cell state in which mitochondria are selected and degraded based on their quality. The observation of secondary mitochondrial particles is reminiscent of mitochondrial-derived vesicles that take part in still poorly characterized pathway of mitochondrial quality control [57,58]. The study of mitochondrial quality control is of great interest, and the approach introduced here has the potential to substantially contribute further to this field by allowing defective mitochondria to be introduced locally in an otherwise functional cellular background or vice versa to study rescue mechanisms of diseased cell states. Moreover, it will be interesting to study the impact of mitochondrial transplantation to the host cells considering metabolic activity and signaling responses. To date, it is not fully clear whether mitochondria transferred in vivo and in vitro settings function as “exchangeable cellular factories” that benefit host cell metabolism as donors of high-quality mitochondrial DNA, as cellular messengers or possibly in combination of the named effects [33–35,59]. Manipulation and observation of mitochondrial subpopulations within a cell are important to drive discoveries in the dynamics, e.g., of asymmetric cell division [3,60]. We show here that subcellular manipulation using FluidFM extends the scope of such studies beyond the application of optogenetic tools and enables the transplantation and observation of tunable quantities of mitochondrial subpopulations in single cells.

We tracked the short-term fate of mitochondrial subpopulations using fluorescent microscopy, but our protocol also allows for the study of the long-term impacts of mitochondrial transfer by showing the introduction of novel mtDNA variants into cell populations. While mitochondrial transfer into oocytes has been demonstrated [61,62], there are only few mechanistic insights into mtDNA selection in eukaryotic cells, with recent notable exceptions [63,64]. Heteroplasmic cells were generated by injection of isolated mitochondria into large oocytes, reaching levels of 7% [64] of transplanted mtDNA under nonselective conditions, which is comparable with our method for somatic cells (up to 2.5%). With a spread from 1 up to about 50 transplanted mitochondria per cell and the opportunity to inject individual cells repeatedly, higher shares of heteroplasmic variants could be achieved. FluidFM thus represents a promising way to decipher modes of mtDNA selection in cell culture models. It will enable the identification of metabolic and genetic factors that impact shifts in mtDNA heteroplasmy and nuclear mitochondrial crosstalk.

In the future, the technique introduced here will stimulate applications in additional research areas, for example, the rejuvenation of cells with low metabolic activity in stem cell therapies [4,65,66] or as an alternative strategy in mitochondrial replacement therapy approaches. Beyond, it offers new perspectives to address fundamental questions in cell biology, mechanobiology, and cell engineering.

## Materials and methods

### Manufacturing process of FluidFM probes with a cylindrical tip

The process starts with a standard 4” silicon wafer in the (100) crystallographic orientation. A 400-nm thick silicon-rich nitride (SiRN) layer is deposited by low pressure chemical vapor deposition (LPCVD) on the selected wafer. The thickness of the SiRN layer determines the thickness of the bottom wall of the microfluidic channel. A silicon oxide layer is deposited by LPCVD from TEOS on the top of the SiRN layer. Then, a circular opening is patterned by reactive ion etching (RIE) on the deposited multilayer. By using deep reactive ion etching (DRIE), a cylindrical pattern is subsequently formed in silicon. The depth of the cylindrical mold defines the height of the cylindrical tip. After the formation of the cylindrical mold, a 150-nm thick SiRN layer is deposited by LPCVD. The thickness of the second SiRN layer determines the thickness of the walls in the cylindrical tip. After the tip molding, a blanket etch (RIE without an etching mask) RIE is performed to remove silicon nitride from the top surface of the wafer and from the bottom of the cylindrical mold. Due to the directionality of the RIE process, the material on the sidewalls is preserved. The silicon oxide layer, which protects the underlying SIRN layer during the RIE etching, is removed. Subsequently, a 1,500-nm thick polysilicon layer is deposited by LOCVD. Polysilicon is used as a sacrificial material to form a microfluidic channel. The thickness of the polysilicon layer determines the height of the microfluidic channel. The layout of the microchannel is patterned by RIE of polysilicon. A SiRN layer with a thickness of 400 nm is deposited by LPCVD after the patterning of polysilicon. This SiRN layer forms the top wall of the fluidic microchannel. The layout of the cantilever and the inlet of the microchannel are defined by RIE of silicon nitride. Next, a reflective metal layer is deposited and patterned on the cantilever. The silicon wafer is anodically bonded to a prediced glass wafer in which the fluidic inlets are premanufactured by powder blasting. After the anodic bonding, the sacrificial polysilicon layer is removed by tetramethylammonium hydroxide (TMAH) etching in order to empty the microchannel and form a hollow cantilever. The bulk silicon is also removed during the TMAH etching to completely release the cantilever. A small part of silicon remains underneath the microfluidic inlet to provide mechanical strength for the channel walls.

### FluidFM probe processing and FIB-SEM imaging and milling

The FluidFM probes were mounted into a custom probe holder and coated with an 18-nm carbon layer using a CCU-010 Carbon Coater (Safematic, Switzerland) before milling by a FIB-SEM Nvision 40 device (Zeiss, Germany) using the Atlas software (Zeiss).

Pyramidal probes used for extraction of organelles and hydrodynamic pulling experiments: The milled face of the pyramidal probe was aligned perpendicularly to the FIB beam equipped with a gallium ion source. Subsequently, the probes were milled with an acceleration voltage of 30 kV at 10 pA until the aperture was milled, controlled by optical observation (approximately to an electric charge of 10 nC per μm^2^). The pyramidal probes have a 100-nm thick silicon nitride layer at the aperture region.

Cylindrical probes used for mitochondrial transplantation experiments were “sharpened” by milling the probe apex at a 50° angle alongside the cylinder at an acceleration voltage of 30 kV at 40 pA. The probes were optically controlled after the milling procedure.

Then, the probes were glued onto a cytoclip holder by Cytosurge (Cytosurge AG, Switzerland). Before each experiment, the cantilevers were cleaned by a 90-second plasma treatment (Plasma Cleaner PDG-32G, Harrick Plasma, USA) before coating overnight with vapor phase SL2 Sigmacote (Sigma-Aldrich, USA) in a vacuum desiccator. The siliconized probe was oven dried at 100 °C for 1 hour. The cantilever spring constant was measured using software-implemented scripts (cylindrical probes: 2 ± 0.4 Nm^−1^, pyramidal probes: 5 ± 1 Nm^−1^).

### FluidFM setup and microscopy

The FluidFM setup is composed of a FlexAFM 5-NIR scan head controlled by a C3000 controller (Nanosurf, Switzerland), a digital pressure controller (ranging from −800 mbar to +1,000 mbar) and Microfluidic Probes (Cytosurge). The scan head is mounted on an inverted AxioObserver microscope equipped with a temperature-controlled incubation chamber (Zeiss). The microscope is coupled to a spinning disk confocal microscope (Visitron, Germany) with a Yokogawa (Japan) CSU-W1 scan head and an EMCCD camera system (Andor, UK). For all images and videos, a 63× oil objective with 1.4 numerical aperture and a 2× lens switcher was used (without lens switcher: 4.85 pixel/micron and 9.69 pixel per micron with lens switcher); images are in 16 bit format. Image acquisition was controlled using the VisiView software (Visitron); linear adjustments and video editing were made with Fiji [67]; additionally, images and videos were noise-filtered using the wiener noise filtering function (wiener2; 3 by 3 neighborhood size) in MATLAB R2018a (MathWorks, USA). Movies were created using a self-written MATLAB script in order to visualize several sections or channels within the same movie. Colormaps originate from Thyng and colleagues [68]. Images of cantilevers containing extracts (Fig 2) were created summing up the slices of a Z-stack via Fiji and reconverting the image to 16 bit format.

### Cell culture

U2OS, COS7, and HeLa cells were maintained in Dulbecco’s Modified Eagle Medium containing 1% penicillin-streptomycin (Thermo Fisher Scientific, USA) and 10% fetal bovine serum (Thermo Fisher Scientific) culture medium at 37 °C and 5% CO_2_ in a humidified incubator. Primary adult HEKa cells were purchased from Thermo Fisher Scientific. HEKa cells were cultivated in EpiLife Medium, with 60 μM calcium, with Human Keratinocyte Growth Supplement, 1%, (Thermo Fisher Scientific) and Gentamicin/Amphotericin Solution (Thermo Fisher Scientific). Cells were seeded 48 hours preceding the experiments onto 50-mm tissue culture treated low μ-dishes (ibidi, Germany) inside 2-well culture inserts (ibidi) or, for experiments coupling mitochondrial extraction via FluidFM to injection of mitochondria, inside 4-well microinserts (ibidi). For the experiments, the culture media was replaced with CO_2_-independent growth medium containing 10% FBS (Thermo Fisher Scientific) and 1% penicillin-streptomycin (Thermo Fisher Scientific). Cell lines stably expressing fluorescent proteins markers were created via lentiviral transduction; the constructs were previously described in Helle and colleagues [52].

### Transient transfection

U2OS-cells were seeded 72 hours preceding the experiment into 2-well culture inserts (ibidi) inside 50-mm tissue culture treated low μ-dishes (ibidi) in a total volume of 100 μL per well. Transfections were performed overnight 36 hours preceding the experiment using 0.2 μg plasmid DNA and 0.2 μL Lipofectamine P3000 solution following the manufacturer’s instructions (Thermo Fisher Scientific). The media was exchanged to standard culture medium 12 hours posttransfection. The plasmid used for calcium imaging CMV-mito-R-GECO1 was a gift from Robert Campbell (Addgene plasmid #46021; http://n2t.net/addgene: 46021; RRID: Addgene_46021).

### Mitochondrial pulling and extraction experiments

All experiments were executed at 37 °C. The cells for extraction/transplantation were selected by optical inspection using light microscopy. Z-stacks were taken before and after the manipulation step to document the workflow. FluidFM probes were prepared as specified above and prefilled with octadecafluorooctane (perfluorooctane) (Sigma-Aldrich). Subsequently, the FluidFM probe was moved over a targeted area in the cytosol of a selected cell, usually close to the nucleus or mitochondrial tubes in the cell periphery. The cantilever was then inserted at the specified location driven by a forward force spectroscopy routine in contact mode, until the set point of 400 nN was reached. The probe was then kept at this position (in the X-Y dimension) at the given force offset. Then, negative pressure in the range between −10 and −150 mbar was applied to aspirate cellular content. Before retracting the probe at the end of the aspiration process, the pressure was set back to 0 mbar. The force set point was adjusted by analyzing force distance curves from neighboring cells within the same experiment; the force value at which the curve takes a linear shape (in this case 80 nN) was estimated. This force value marks the point at which the probe makes contact with the glass bottom below the cell; consequently, this value was chosen as a set point for the extraction. Extraction of the ER fraction: The experiments were performed using pyramidal cantilevers featuring an aperture area of 0.5 μm^2^ (see Fig 1B) at −20 mbar using U2OS or COS7 cells. Experiments for both cell lines were repeated twice on different days. Extraction of mitochondria and mitochondria pulling experiments were performed using pyramidal cantilevers featuring an aperture area of 1 μm^2^ or cantilevers with a cylindrical apex featuring an aperture area of 1.5 μm^2^ (see Fig 1B) at −20 to −100 mbar using U2OS or HeLa cells. Experiments including extraction of mitochondria were executed in 32 individual experimental setups on 32 individual days. Experiments showing recruitment of Drp1 were repeated 4 times on 4 different days with at least 5 cells per experiment in U2OS cells. Mitochondrial pulling experiments connected with calcium imaging and thapsigargin treatment (Fig 5) were executed 3 times, each time on an individual day with at least 7 cells per experiment.

For probe insertion with minimal Ca^2+^ influx as shown in S7 Movie, newly coated (Sigmacote; see above) FluidFM cantilevers were used. To deliberately disturb the cell membrane (S9 Movie), the probe was driven into the cell using the same set point, then the optical table (Newport, USA) was gently flicked with the index finger to cause the probe to shift slightly, effectively disturbing the cell membrane. Cell viability was controlled using the LIVE/DEAD cell imaging kit (Thermo Fisher Scientific).

### Mitochondrial transplantation experiments

FluidFM injection of bulk-purified mitochondria: Mitochondria were purified from approximately 2*10^6^ cultured HeLa cells continuously expressing the mitochondrial matrix marker su9-mCherry using the Qproteome Mitochondria Isolation Kit (Qiagen, Germany) following the manufacturer’s instructions. After purification, the mitochondria were washed 2 times in injection buffer (see above) and finally resuspended in 40-μl injection buffer before being loaded into FluidFM probes having a sharpened cylindrical apex.

Coupling mitochondrial extraction with transplantation from individual cell to cell: Mitochondria were aspired as described above, using FluidFM probes having a sharpened cylindrical apex from HeLa-cells that were cocultured on the same dish, previously seeded within another quadrant of the 4-well micro insets. Subsequently, the probe was moved to a region containing cells targeted for transplantation. For injection of mitochondria, the probe was positioned above a target cell as described above for the extraction of organelles. The cantilever containing the organelles was then inserted into the cell in contact mode (set point 100 nN). The injection process was controlled by observing the process in brightfield in real time, at first, a positive pressure of 20 mbar was applied and the separation line between extract and perfluorooctane was monitored, the pressure was increased until the line was moving toward the aperture (maximal value of 200 mbar), effectively pushing the cell extract into the recipient cell. When the extract was totally or in large parts transferred to the recipient cell, the pressure was then set to 0 mbar, and the probe was retracted. Z-stacks were taken to control for successfully transferred mitochondria. Cell viability was controlled using the LIVE/DEAD cell imaging kit (Thermo Fisher Scientific) following the manufacturer’s instruction. Both experimental approaches were performed in 6 individual experiments, each on individual days.

### Quantification of genetic incorporation of transplanted mitochondria

All experiments were conducted in biological triplicates. (A) FluidFM injection of purified mitochondria: Single HeLa cells continuously expressing the mitochondrial matrix marker su9-BFP were seeded into a single quadrant of a 4-well microinsert (ibidi) 72 hours before the experiment, at the day of the experiment, each seeded well contained 4 to 6 cells. All cells were injected as described above. (B) Coupling mitochondrial extraction with transplantation from individual cell to cell: One quadrant of a 4-well microinsert (ibidi) was seeded with recipient HeLa (su9-BFP) cells as described above. A second quadrant was seeded with U2OS cells continuously expressing su9-mCherry as donors. The transplantation was executed as described above. Subsequently, the U2OS cells were removed, again using a FluidFM probe filled with 1% sodium dodecyl sulfate (Merck Group, Germany). Before sequencing, the whole cell population was controlled for any signal mCherry positive cells via fluorescence activated cell sorting, but no unwanted carryover of remaining U2OS cells (mCherry+) could be detected. Control for nonspecific mitochondrial carryover: Approximately 100,000 Hela cells expressing the mitochondrial matrix marker su9-BFP were seeded onto a culture dish. They were mixed with purified mitochondria from approximately 2*10^6^ U2OS cells expressing su9-mCherry by pipetting the extracts gently on top of the cultured cells. Cells obtained after treatments A and B were grown for 8 days, transferred into a new culture dish and grown for another 6 days. The control cells were grown for 3 days, transferred into a new culture dish and grown for another 3 days. Subsequently, the total DNA of all samples was purified using the MasterPure Complete DNA and RNA Purification Kit (epicenter) following the manufacturer’s instructions. Part of the D-loop region was amplified via PCR using primers 1 and 2 (S2 Table). To create a “null hypothesis” for the following sequencing workflow, 3 test samples were created consisting of 0.1%/0.5%/1% U2OS amplicons, mixed with HeLa amplicons. Subsequently, all samples were sequenced using the a Pacbio Sequel SMRT cell, subsequently analyzed as described in Russo and colleagues [69] The reads were assigned to either U2OS mtDNA or Hela mtDNA using 4 conserved mutations of U2OS cells within this region previously identified via Sanger sequencing (15959G>T, 16069C>T, 16108C>T, 16126T>C; see S1 Table).

### Statistics and reproducibility

All representative experiments were repeated at least 3 times independently with similar results. Absolute counts of cells or mitochondrial particles are included in the main text or in the figure legends. Analysis for single nucleotide polymorphisms was performed as described in Russo and colleagues [69]. Performed statistical tests and significance levels are indicated in the figures and respective figure legends. Statistical analyses can be found below the plotted data in S1 Data.

### Analysis of mitochondrial quality control mechanisms with mitochondrial transplantation in primary HEKa cells

Cells were seeded into as described above, into low μ-dishes (ibidi) inside 2-well culture inserts (ibidi). The special separation allows for drug-treatments of the donor cell population before the experiment, while the host cell population of HEKa cells remains under standard culture conditions. At the beginning of the experiment, the cell media was replaced with CO_2_-independent growth medium containing 10% FBS (Thermo Fisher Scientific) and 1% penicillin-streptomycin (Thermo Fisher Scientific). A total of 4 μM oligomycin was added when indicated. Treatment conditions: 10 μM CCCP for 3 hours, 8 μM doxycycline for 48 hours, 750 μM H_2_O_2_ for 3 hours. Mitochondria were extracted from Hela cells and transplanted into HEKa cells. A Z-stack image series of the transplant and the host cell was taken directly after the transplantation process with a 63× oil objective with 1.4 numerical aperture and a 2× lens switcher in 500-nm steps. Further Z-stacks were taken at other time points depending on the individual experiment. For the endpoint at 18 to 22 hours posttransplantation, the mitochondrial network was visualized using MitoTracker Green FM (Thermo Fisher Scientific) following the manufacturer’s instructions. The overlap between the transplant and the host mitochondrial network was controlled. For the quantitative analysis, data were analyzed with self-written MATLAB (R2018a) scripts.

### Split-kinesin experiments

pKIF5C-HA-FRB was a kind gift from Prof. Sean Munro. Expresses fusion of Rat kinesin minus tail to FRB; pFKBP-mCh-Fis1TM was subcloned from pFKBP-GFP-myc-GRIP (Sean Munro) by swapping the GFP-myc-GRIP fragment with mCherry fused to the TM domain of yeast Fis1 for OMM targeting. U2OS cells stably expressing mtBFP and a shRNA against DRP1 [52] were reverse transfected with the 2 constructs above. Two days later, cells were imaged using a spinning disk microscope. Rapamycin was diluted in growth medium to a final concentration of 1 nM. A microfluidic imaging device was used to allow (rapamycin containing) medium replacement while imaging. Experiments were performed twice on different days.

## Supporting information

S1 Fig Cantilever fabrication and FluidFM setup. **(A)** FluidFM fabrication process. (**a**–**n**) Steps for the fabrication of hollow FluidFM cantilevers comprising a cylindrical apex. (**B**) Schematic overview of the FluidFM setup.
(PDF)

S2 Fig Mechanical robustness of FluidFM cantilevers. Mechanical robustness of FluidFM cantilevers comprising a sharpened cylindrical apex. FIB images of FluidFM cantilevers: (**A**) Side view of a slanted cylindrical FluidFM cantilever with a 1.2-μm diameter of the cylinder. (**B**) Cantilever that was used for mitochondrial transplantation with a set point of 1,000 nN, the apex is broken impairing insertion of the probe into cells. (**C**) Cantilever that was used for mitochondrial transplantation with a set point of 400 nN, the apex remains intact. Scale bar: 10 μm. FIB, focused ion beam.
(PDF)

S3 Fig ER extraction of COS7-cells. ER extraction of COS7-cells. COS7-cells stably expressing the ER membrane marker Sec61-GFP and mitochondrial matrix marker su9-BFP. Small square indicates cantilever position, and big, dashed line indicates a zone of ER rearrangement. Scale bar: 5 μm. ER, endoplasmic reticulum.
(PDF)

S4 Fig Viability of Hela cells postextraction of mitochondria. Viability of Hela cells postextraction of mitochondria. (**A**) Overview of HeLa cells 2 hours postmitochondrial extraction, stained with the LIVE-DEAD Cell imaging kit. Viable cells show green fluorescence signal, dead cells show red fluorescence. Areas 1 to 5 show regions with extracted cells. Scale bar: 50 μm. (**B**) Mitochondrial networks (su9-BFP) from the regions shown in a, before and 2 hours postextraction. The insertion sites of the cantilevers for extraction are highlighted by blue circles. The experiment was conducted twice, 36 out of 37 sampled cells remained viable. Scale bar: 10 μm.
(PDF)

S5 Fig Mitochondrial shape transition and fission. **(A)** Schematic representation of the working model of the shape transition into the pearls-on-a-string phenotype upon exertion of pulling forces. Small box icons represent cantilever apexes applying negative hydrodynamic forces (−Δp), or without pressure differences (Δp = 0). (**B**) Images of U2OS cells expressing Fis1TM-mCherry (OMM, gray) and su9-BFP (mitochondrial matrix, red) during membrane pulling via FluidFM. Yellow triangle indicates position of the aperture inside the cell. 0 second, Δp = 0 mbar. 1 second to 4 seconds, Δp = -50 mbar. Scale bar: 5 μm (left image), 2 μm (images on the right). (**C**) Time-lapse of mitochondrial network upon pulling. Arrowheads indicate sites of a fission event. Scale bar: 10 μm. See also S5 Movie. (**D)** Image series U2OS-cells overexpressing Drp1-mCherry (cyan) and quantified fluorescent signal along mitochondrial tubes during force induced shape transition of pulled mitochondria. (**E**) U2OS cell expressing kinesin-FRP (minus tail) and FKBP-Fis1. Mitochondria fragment following addition of rapamycin. Scale bars: 10 μm. The data underlying S5D Fig can be found in S1 Data. Drp1, dynamin-related protein 1; OMM, outer mitochondrial membrane.
(PDF)

S6 Fig Calcium imaging of pearls-on-a-string transition. Mitochondrial shape transition is Ca^2+^-independent. (**A**, **B**) Ca^2+^ imaging series of mitochondrial shape changes. U2OS cells express su9-BFP (mitochondrial matrix, cyan) and the Ca^2+^-sensing fluorophore mito-R-GECO1 (red). The upper panel shows an overlay of the 2 fluorophores during extraction. The middle panel shows an enlarged section of (left panel) a mitochondrion directly adjacent to the cantilever tip and (right panel) a peripheral mitochondrion. The bottom panel shows the total fluorescence intensity of mito-R-GECO1 of the displayed mitochondria during the manipulation process. Yellow boxes indicate position of the cantilever aperture, arrows indicate time point of application of −Δ*p*. (**A**) Experiment done in culture medium containing 0.7 μM calcium. (**B**) Experiment executed in culture medium after addition of 2 μM EGTA. (**C**) Pulling experiment of an individual mitochondrial tube 30 minutes after the addition of 5 μM thapsigargin. *n* = 8. Scale bars: 10 μm. The data underlying S6A and S6B Fig can be found in S1 Data.
(PDF)

S7 Fig Hela cells postmitochondrial transplantation. (**A**) Images of Hela cells postmitochondrial transplantations via the cell-to-cell and the injection approach. Images show and overlay of brightfield (gray) and the transplant (su9-mCherry, red). Transplanted cells are outlined in yellow. (**B**) Fluorescence-microscopy images of bulk-extracted mitochondria with labeled matrix (su9-BFP, blue) and labeled outer membrane (Fis1TM-mCherry, red). Yellow arrows indicate mitochondrial particles showing both, matrix and outer membrane labels. White arrows indicate mitochondrial particles missing the outer membrane. Scale bars: 10 μm.
(PDF)

S8 Fig Drug treatments of HEKa and Hela cells. Fusion states of the mitochondrial network of HEKa cells upon drug-treatments. (**A**) HEKa cells in culture medium, the mitochondrial network is visualized using mitoTracker Green. Cells in the lower panel were treated with 4 μM oligomycin for 22 hours. (**B**) Hela cells in culture medium, the mitochondrial matrix is visualized via permanent expression of su9-mCherry. Treatments: 10 μM CCCP for 3 hours, 8 μM doxycycline for 24 hours, 750 μM H_2_O_2_ for 3 hours. Scale bars: 10 μm. HEKa, human endothelial keratinocyte.
(PDF)

S9 Fig Transplant uptake in Hela cells. Visualization and analysis of transplanted mitochondria in single Hela cells. Top: surface render of the total fluorescence of the transplant over time bottom: objects plotted by size in pixel and normalized fluorescence intensity. Objects carrying the high fluorescence intensity of the initial transplant at the 0 hour time point are depicted in red; objects with low fluorescence are depicted in gray. (**A**) Hela cell showing mitochondrial acceptance of 8 mitochondria within 2 hours. (**B**) Hela cell showing full degradation of the transplant, 7 mitochondria were transplanted. The data underlying S9A and S9B Fig can be found in S1 Data.
(PDF)

S10 Fig Transplant degradation on HEKa cells. Exemplary quantification of mitochondrial degradation in HEKa cells. (**A**) Fluorescence microscopy images of a cell 19 hours postmitochondrial transplantation, 4 mitochondria were initially transplanted. The enlarged section shows a presumptive mitophagosomal structure. Scale bar: 10 μm. (**B**) Total number of fluorescent objects within the host cell over time. (**C**) Time-lapse images of mitochondrial degradation. Surface-rendered images of all detected fluorescent images show the increasing number and spatial distribution of particles over time. Scale bar: 10 μm. (**D**) Scatter plots of objects plotted by size in pixel (logarithmic scale) and normalized fluorescence intensity. Outlined dot shows the largest tertiary particle shown in the central panel in c. **e**, degradation of individual mitochondria over time [hour]. After fusion with a presumptive mitophagosomal structure at 4:40 hours, the degradation progresses for over 8 hours. Scale bar: 1 μm. The data underlying S10B and S10D Fig can be found in S1 Data. HEKa, human endothelial keratinocyte.
(PDF)

S11 Fig Mitochondrial transplant fluorescence over time. Fluorescent traces of the mitochondrial transplant and directly derived particles in single HEKa cells over time. Plots show the total volume occupied by unfused mitochondrial transplant over time in various conditions. The number of initially transplanted mitochondria per cell is depicted on the upper right. (**A**) Transplant was treated with CCCP and oligomycin. (**B**) Transplant was treated with doxycycline, CCCP, and oligomycin. (**C**) Transplant was treated with doxycycline. The data underlying S11A–S11C Fig can be found in S1 Data. HEKa, human endothelial keratinocyte.
(PDF)

S12 Fig Transplant fate in HEKa cells. (**A**) Fusion and degradation behavior of drug-compromised mitochondrial transplants in HEKa cells. Each condition was tested with 22 cells. **(B**) Distribution of mitochondrial quantity transplanted per cell from all conditions tested in a (22 cells for each conditions). Line shows median value. The data underlying S12A and S12B Fig can be found in S1 Data. HEKa, human endothelial keratinocyte.
(PDF)

S13 Fig p55 nucleoid traces. Tracing of p55 nucleoids overlap with mitochondria during extraction via FluidFM. (**A**, **B**) Traces of separated mitochondria (su9-BFP) showing overlap with p55-GFP signals in red and of separated mitochondria showing no traces of p55-GFP in blue. White arrowhead indicates cantilever apex position. Panels below show the extraction sites comprising the area used for data acquisition. The mitochondrial network fluorescence is shown in blue, p55-GFP speckles are shown in red. Scale bars: 5 μm. (**C**) Traces of all cells analyzed in this manner. The data underlying S13A–S13C Fig can be found in S1 Data.
(PDF)

S1 Movie Organelle extraction using FluidFM. Brightfield video of HEK cells being extracted with a FluidFM cantilever prefilled with perfluorooctane. Scale bar: 10 μm.
(AVI)

S2 Movie ER extraction from a COS7 cell using FluidFM. Panels from left to right: Mitochondrial matrix, su9-BFP; ER, Sec61-GFP; Merge of panels 1 and 2: mitochondria (red) and ER (cyan). Arrowheads mark the site of extraction. Scale bar: 5 μm. ER, endoplasmic reticulum.
(AVI)

S3 Movie Cell-wide view of ER extraction from a COS7 cell using FluidFM. Channels from left to right: Mitochondrial matrix, su9-BFP; ER, Sec61-GFP; merge of channels 1 and 2: mitochondria (red) and ER (cyan). Arrowheads mark the site of extraction. Scale bars: 10 μm. ER, endoplasmic reticulum.
(AVI)

S4 Movie Extraction of mitochondria from a U2OS cells using FluidFM. Mitochondrial matrix is labeled via su9-BFP. Arrowhead indicates site of extraction. Left side: cell wide view of the extraction process; scale bar: 10 μm. Right side: enlarged view of the extraction site; scale bar: 5 μm.
(AVI)

S5 Movie Extraction of singular mitochondrial sphere from a U2OS cell using FluidFM. Mitochondrial matrix is labeled via su9-BFP. Arrowhead indicates site of extraction. Left side: cell wide view of the extraction process; scale bar: 10 μm. Right side: enlarged view of the extraction site; scale bar: 5 μm.
(AVI)

S6 Movie Pearling of mitochondrial tubes upon exertion of pulling force. Channels from left to right: Mitochondrial matrix: su9-BFP; OMM: Fis1TM-mCherry; merge of mitochondrial matrix (red) and OMM (cyan). Arrowheads indicate site of extraction. Scale bars: 5 μm. OMM, outer mitochondrial membrane.
(AVI)

S7 Movie Calcium imaging during mitochondrial extraction without membrane disruption via FluidFM. Movie of an individual U2OS cell. Channels from left to right: Ca^2+^ sensor within the mitochondrial matrix, mito-R-GECO1; mitochondrial matrix, su9-BFP; merge of the Ca^2+^ sensor (red) and the mitochondrial matrix label (cyan). Arrowheads indicate site of extraction. Scale bar: 10 μm.
(AVI)

S8 Movie Calcium imaging during membrane disruption via FluidFM. Movie of the individual U2OS cell shown in S7 Movie. Channels from left to right: Ca^2+^ sensor within the mitochondrial matrix, mito-R-GECO1; mitochondrial matrix, su9-BFP; merge of the Ca^2+^ sensor (red) and the mitochondrial matrix label (cyan). Arrowhead indicates site of extraction. Scale bar: 10 μm.
(AVI)

S9 Movie Calcium imaging during extraction with mild membrane disruption. Channels from left to right: Ca^2+^ sensor within the mitochondrial matrix, mito-R-GECO1; mitochondrial matrix, su9-BFP; merge of the Ca^2+^ sensor (red) and the mitochondrial matrix label (cyan). Arrowheads indicate site of extraction. Scale bars: 10 μm.
(AVI)

S10 Movie Calcium imaging during extraction with mild membrane disruption after addition of EGTA. Channels from left to right: Ca^2+^ sensor within the mitochondrial matrix, mito-R-GECO1; mitochondrial matrix, su9-BFP; merge of the Ca^2+^ sensor (red) and the mitochondrial matrix label (cyan). Arrowheads indicate site of extraction. Scale bars: 10 μm.
(AVI)

S11 Movie Fusion of a singular transplanted mitochondrial sphere with the mitochondrial network of a U2OS cell. Channels from left to right: Mitochondrial matrix of the transplanted mitochondrion, su9-mCherry; mitochondrial matrix of the host cell network, su9-BFP; merge: transplant mitochondria (red) and host network (cyan). Scale bar: 10 μm.
(AVI)

S12 Movie Time-lapse images of mitochondrial acceptance in HEKa cells. Left: Brightfield images. Right: Fluorescence signal of the transplanted mitochondria, su9-mCherry in the ’hot’ colormap, MATLAB R2018a, 8bit. The amount of the initially transplanted mitochondria are depicted in the first and last frame next to the respective cell. HEKa, human endothelial keratinocyte.
(AVI)

S1 Text Force-induced mitochondrial fission. (DOCX)

S1 Data Data underlying figures. (XLSX)

S1 Table Viability of HeLa cells post mitochondrial transplantation: Injection of purified mitochondria, extracted from bulk cultured cells. (PDF)

S2 Table Primers used in this study. (PDF)

## Abbreviations

AFM: atomic force microscope
DRIE: deep reactive ion etching
Drp1: dynamin-related protein 1
ER: endoplasmic reticulum
FIB: focused ion beam
HEKa: human endothelial keratinocyte
LPCVD: low pressure chemical vapor deposition
mtDNA: mitochondrial DNA
OMM: outer mitochondrial membrane
RIE: reactive ion etching
SiRN: silicon-rich nitride
TMAH: tetramethylammonium hydroxide
