# Supplementary material for: Mitochondria transplantation between living cells
Source: PLoS Biol. 2022 Mar 23;20(3):e3001576. doi: 10.1371/journal.pbio.3001576 (PMC8942278; doi:10.1371/journal.pbio.3001576)
Supplement: S1 Fig — (A) FluidFM fabrication process. (a–n) Steps for the fabrication of hollow FluidFM cantilevers comprising a cylindrical apex. (B) Schematic overview of the FluidFM setup. (PDF) [file pbio.3001576.s001.pdf]

A

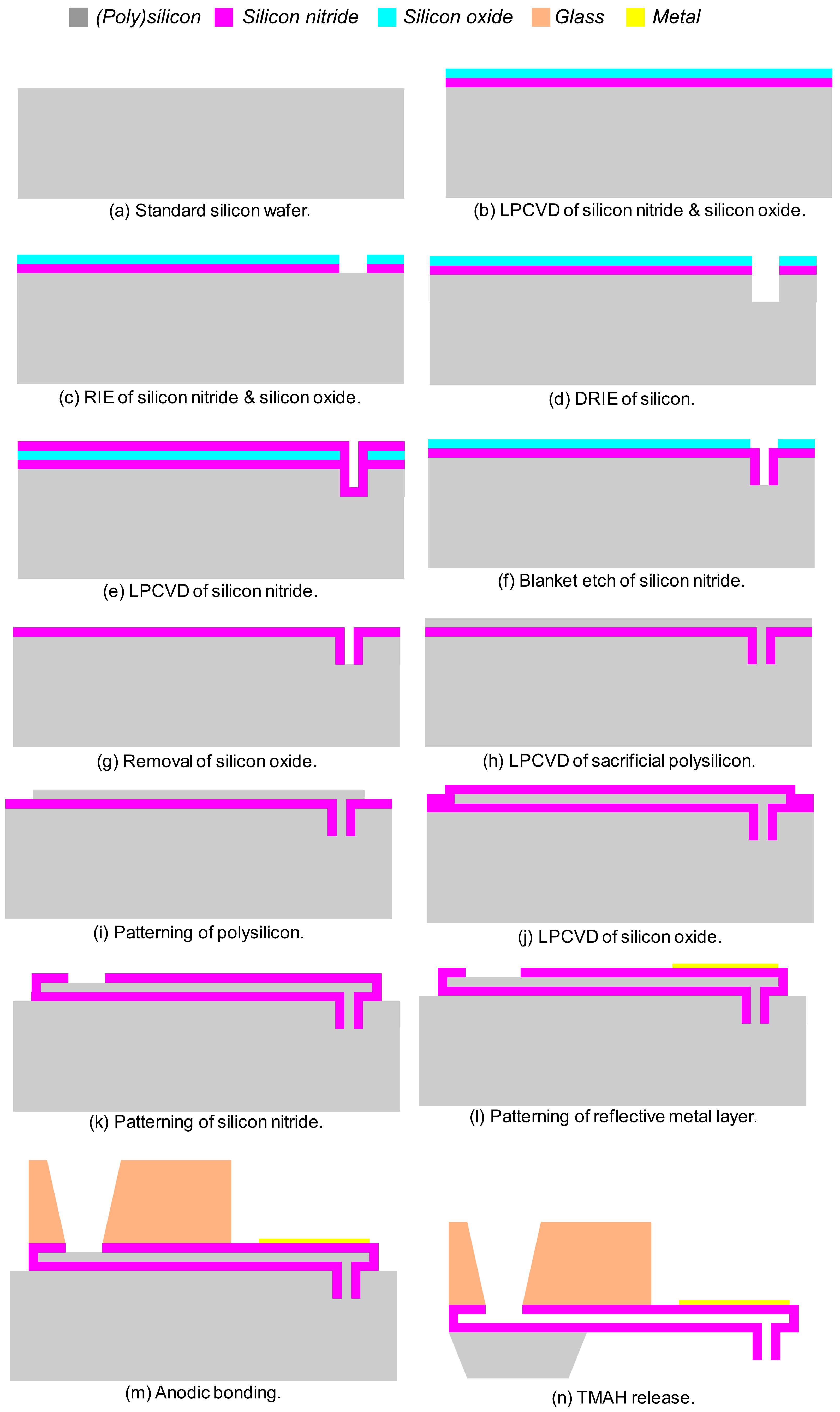
(Poly)silicon
Silicon nitride
Silicon oxide
Glass
Metal
(a) Standard silicon wafer.
(b) LPCVD of silicon nitride & silicon oxide.
(c) RIE of silicon nitride & silicon oxide.
(d) DRIE of silicon.
(e) LPCVD of silicon nitride.
(f) Blanket etch of silicon nitride.
(g) Removal of silicon oxide.
(h) LPCVD of sacrificial polysilicon.
(i) Patterning of polysilicon.
(j) LPCVD of silicon oxide.
(k) Patterning of silicon nitride.
(l) Patterning of reflective metal layer.
(m) Anodic bonding.
(n) TMAH release.

B

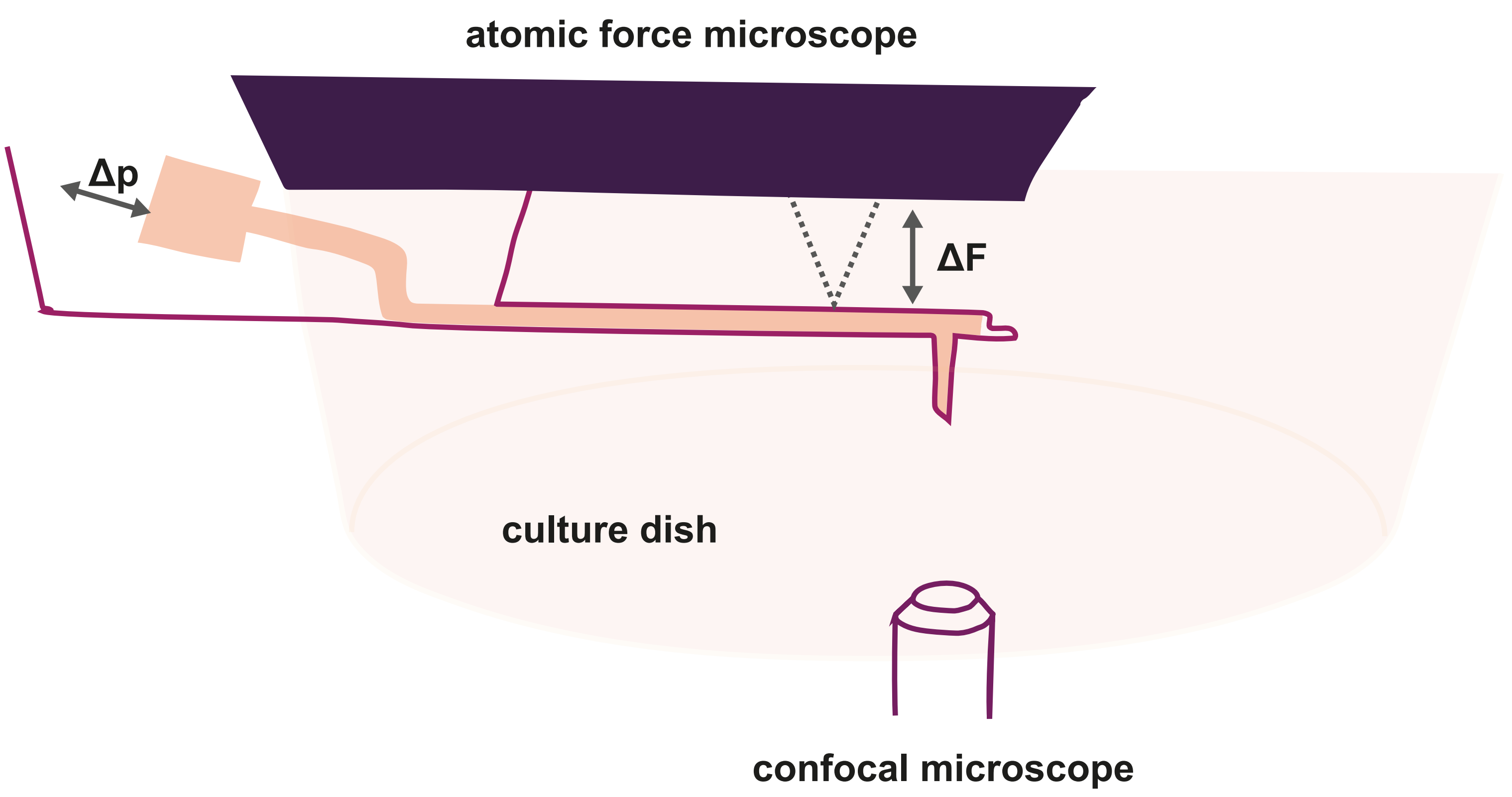
atomic force microscope
Δp
ΔF
culture dish
confocal microscope
