# Supplementary figures and images for: Mitochondria transplantation between living cells
Source: PLoS Biol. 2022 Mar 23;20(3):e3001576. doi: 10.1371/journal.pbio.3001576 (PMC8942278; doi:10.1371/journal.pbio.3001576)

A

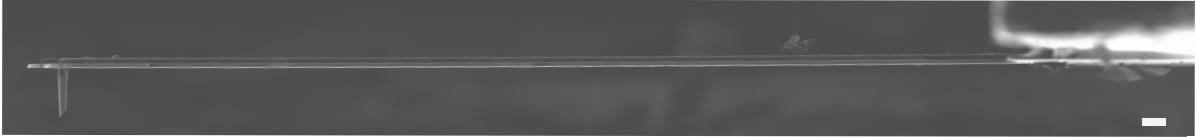

B

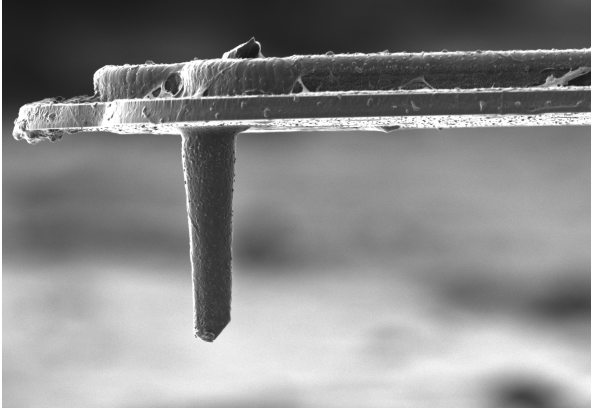

C

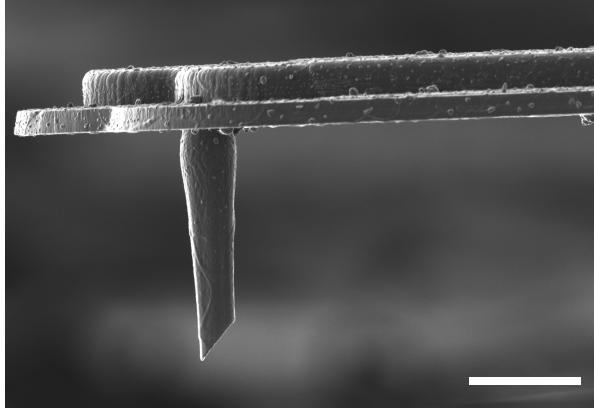

Supplement: S2 Fig — Mechanical robustness of FluidFM cantilevers comprising a sharpened cylindrical apex. FIB images of FluidFM cantilevers: (A) Side view of a slanted cylindrical FluidFM cantilever with a 1.2-μm diameter of the cylinder. (B) Cantilever that was used for mitochondrial transplantation with a set point of 1,000 nN, the apex is broken impairing insertion of the probe into cells. (C) Cantilever that was used for mitochondrial transplantation with a set point of 400 nN, the apex remains intact. Scale bar: 10 μm. FIB, focused ion beam. (PDF) [file pbio.3001576.s002.pdf]

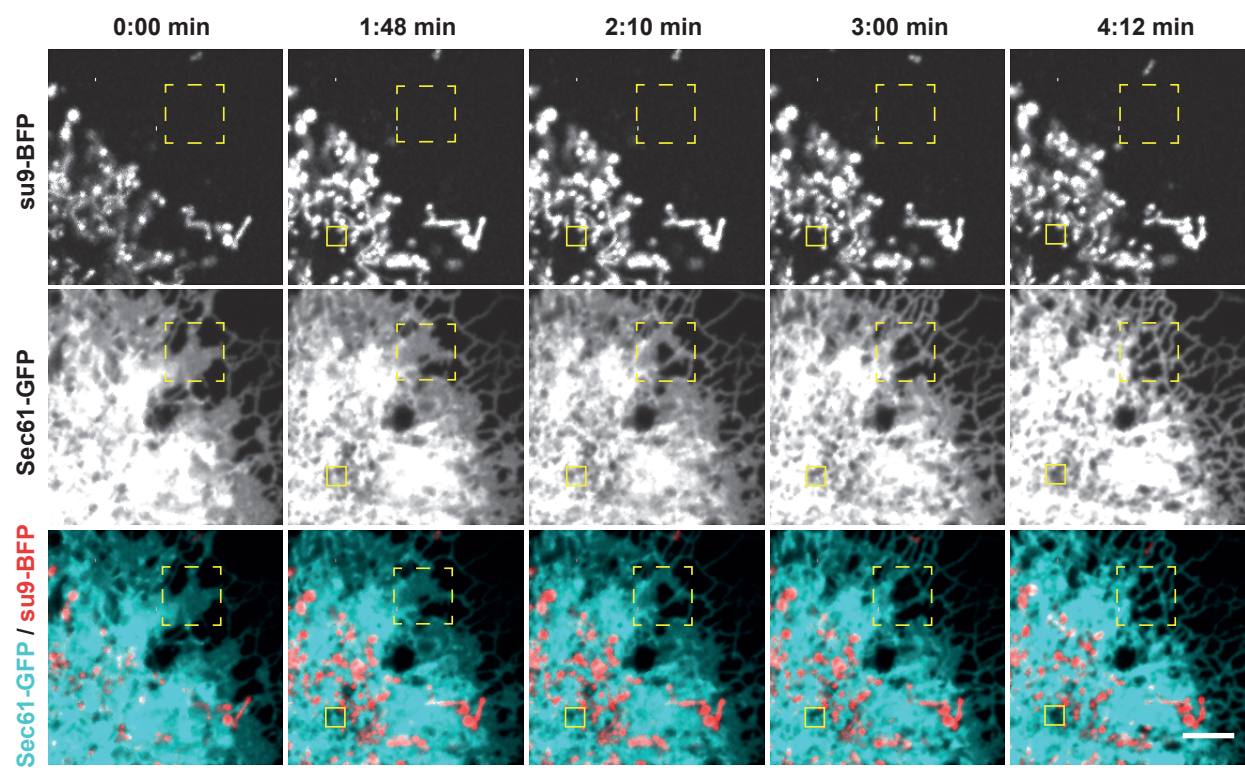

Supplement: S3 Fig — ER extraction of COS7-cells. COS7-cells stably expressing the ER membrane marker Sec61-GFP and mitochondrial matrix marker su9-BFP. Small square indicates cantilever position, and big, dashed line indicates a zone of ER rearrangement. Scale bar: 5 μm. ER, endoplasmic reticulum. (PDF) [file pbio.3001576.s003.pdf]

A

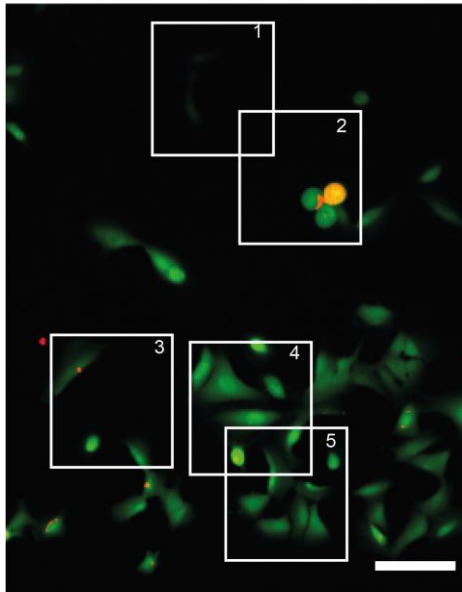

B

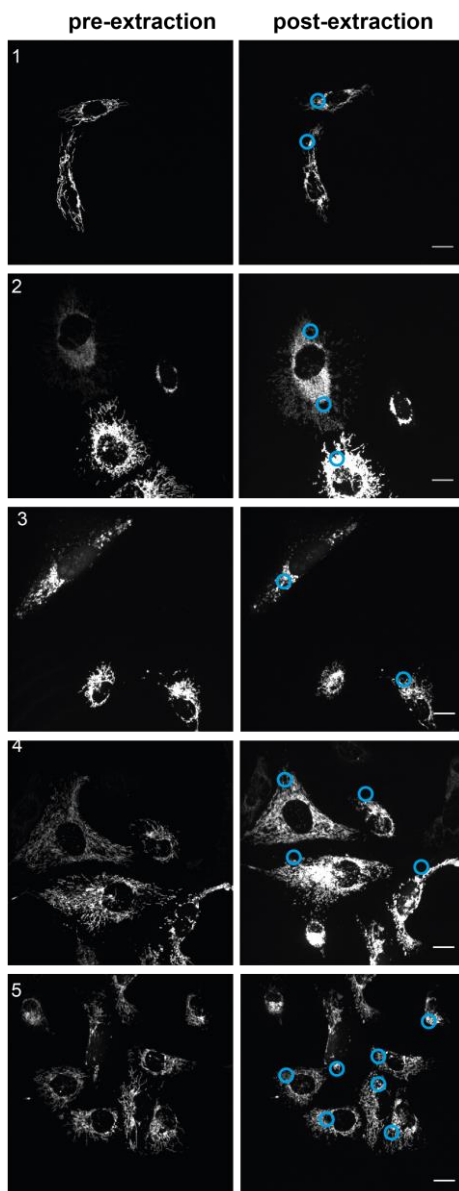

Supplement: S4 Fig — Viability of Hela cells postextraction of mitochondria. (A) Overview of HeLa cells 2 hours postmitochondrial extraction, stained with the LIVE-DEAD Cell imaging kit. Viable cells show green fluorescence signal, dead cells show red fluorescence. Areas 1 to 5 show regions with extracted cells. Scale bar: 50 μm. (B) Mitochondrial networks (su9-BFP) from the regions shown in a, before and 2 hours postextraction. The insertion sites of the cantilevers for extraction are highlighted by blue circles. The experiment was conducted twice, 36 out of 37 sampled cells remained viable. Scale bar: 10 μm. (PDF) [file pbio.3001576.s004.pdf]

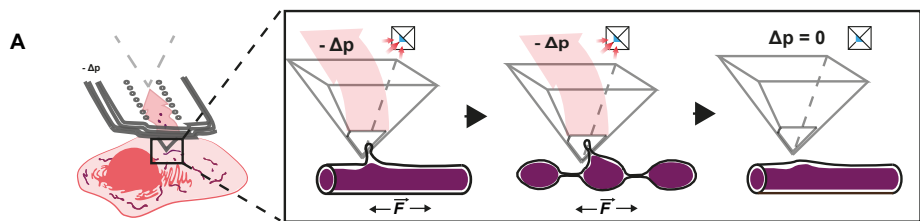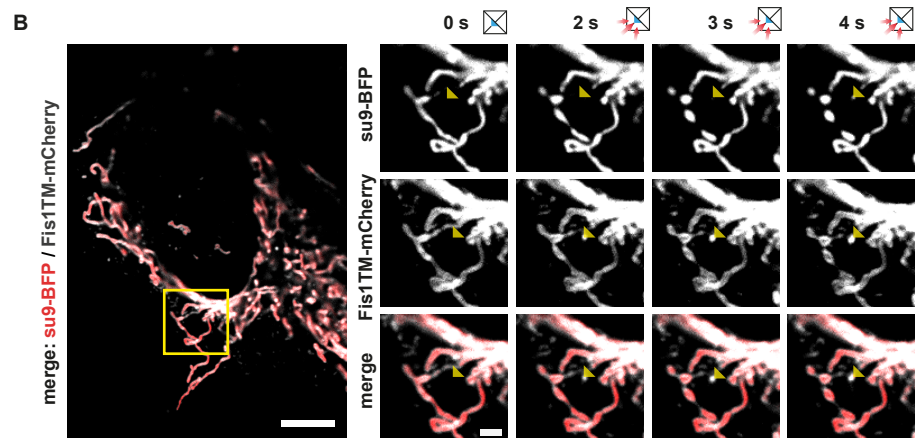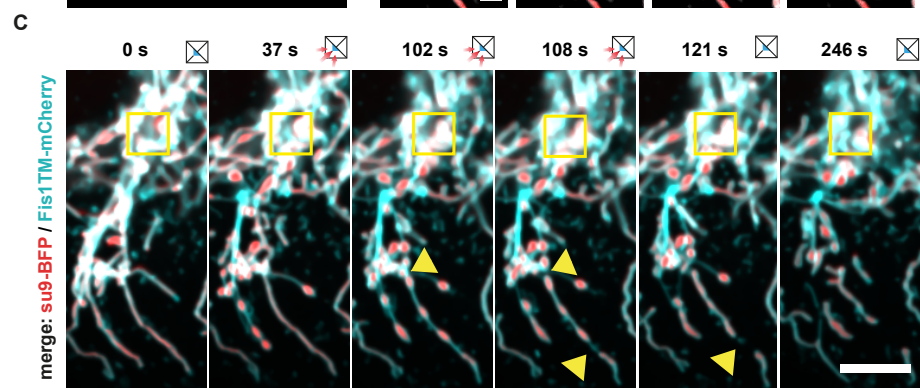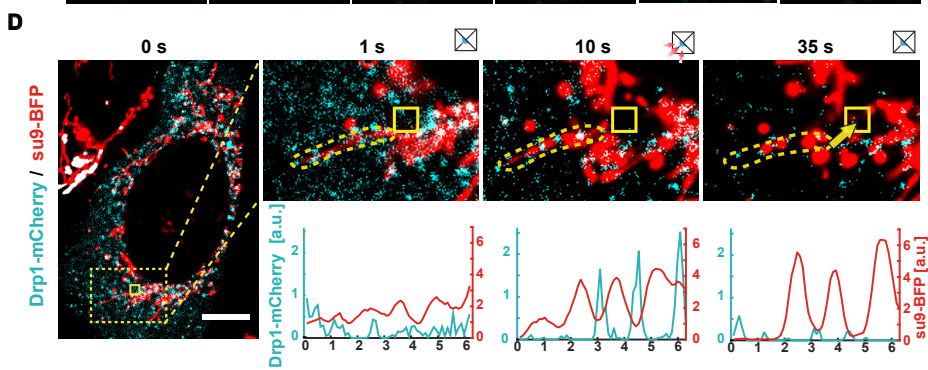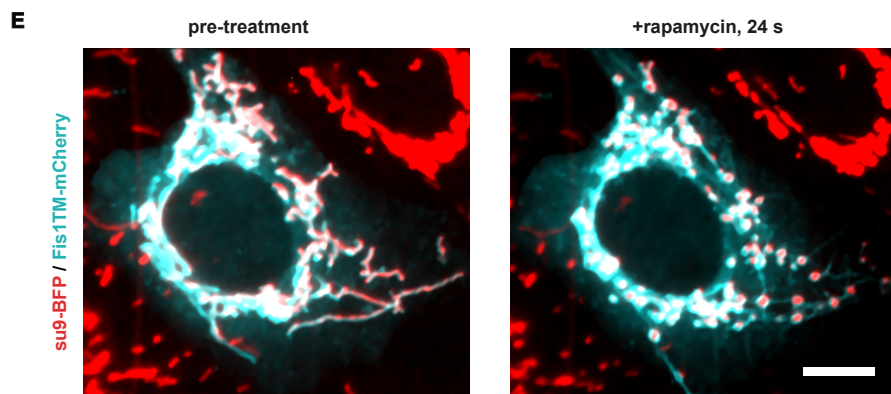

Supplement: S5 Fig — (A) Schematic representation of the working model of the shape transition into the pearls-on-a-string phenotype upon exertion of pulling forces. Small box icons represent cantilever apexes applying negative hydrodynamic forces (−Δp), or without pressure differences (Δp = 0). (B) Images of U2OS cells expressing Fis1TM-mCherry (OMM, gray) and su9-BFP (mitochondrial matrix, red) during membrane pulling via FluidFM. Yellow triangle indicates position of the aperture inside the cell. 0 second, Δp = 0 mbar. 1 second to 4 seconds, Δp = -50 mbar. Scale bar: 5 μm (left image), 2 μm (images on the right). (C) Time-lapse of mitochondrial network upon pulling. Arrowheads indicate sites of a fission event. Scale bar: 10 μm. See also S5 Movie. (D) Image series U2OS-cells overexpressing Drp1-mCherry (cyan) and quantified fluorescent signal along mitochondrial tubes during force induced shape transition of pulled mitochondria. (E) U2OS cell expressing kinesin-FRP (minus tail) and FKBP-Fis1. Mitochondria fragment following addition of rapamycin. Scale bars: 10 μm. The data underlying S5D Fig can be found in S1 Data. Drp1, dynamin-related protein 1; OMM, outer mitochondrial membrane. (PDF) [file pbio.3001576.s005.pdf]

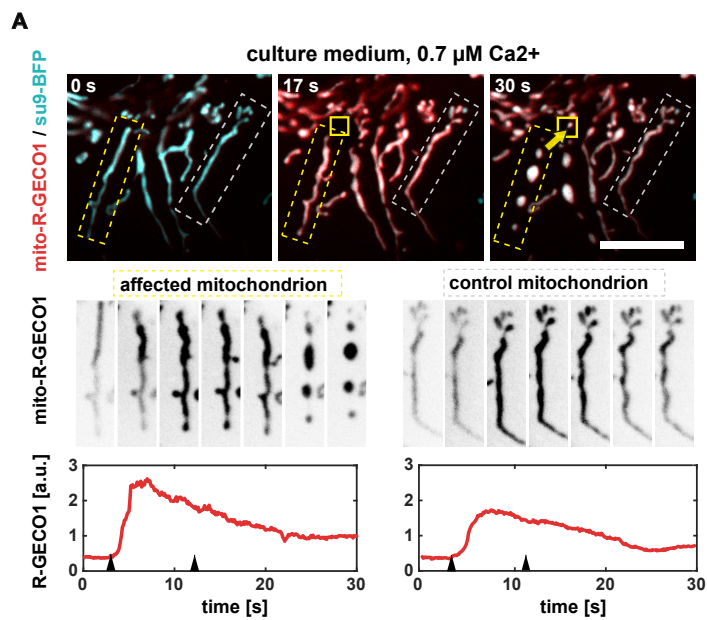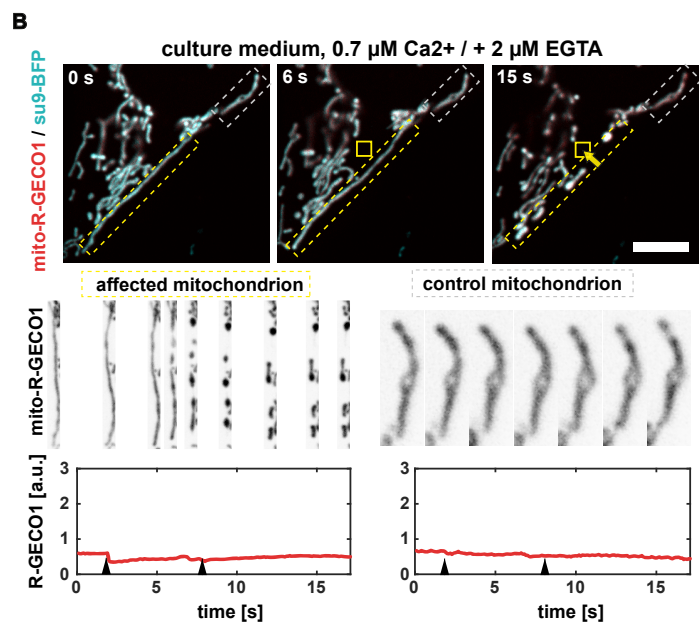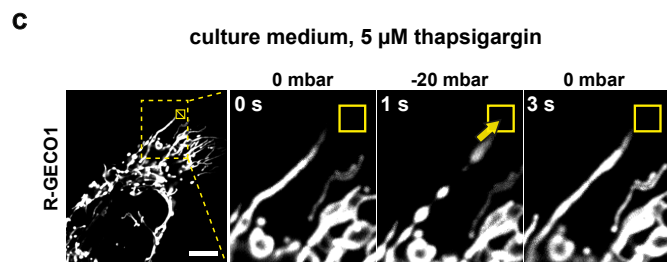

Supplement: S6 Fig — Mitochondrial shape transition is Ca2+-independent. (A, B) Ca2+ imaging series of mitochondrial shape changes. U2OS cells express su9-BFP (mitochondrial matrix, cyan) and the Ca2+-sensing fluorophore mito-R-GECO1 (red). The upper panel shows an overlay of the 2 fluorophores during extraction. The middle panel shows an enlarged section of (left panel) a mitochondrion directly adjacent to the cantilever tip and (right panel) a peripheral mitochondrion. The bottom panel shows the total fluorescence intensity of mito-R-GECO1 of the displayed mitochondria during the manipulation process. Yellow boxes indicate position of the cantilever aperture, arrows indicate time point of application of −Δp. (A) Experiment done in culture medium containing 0.7 μM calcium. (B) Experiment executed in culture medium after addition of 2 μM EGTA. (C) Pulling experiment of an individual mitochondrial tube 30 minutes after the addition of 5 μM thapsigargin. n = 8. Scale bars: 10 μm. The data underlying S6A and S6B Fig can be found in S1 Data. (PDF) [file pbio.3001576.s006.pdf]

A

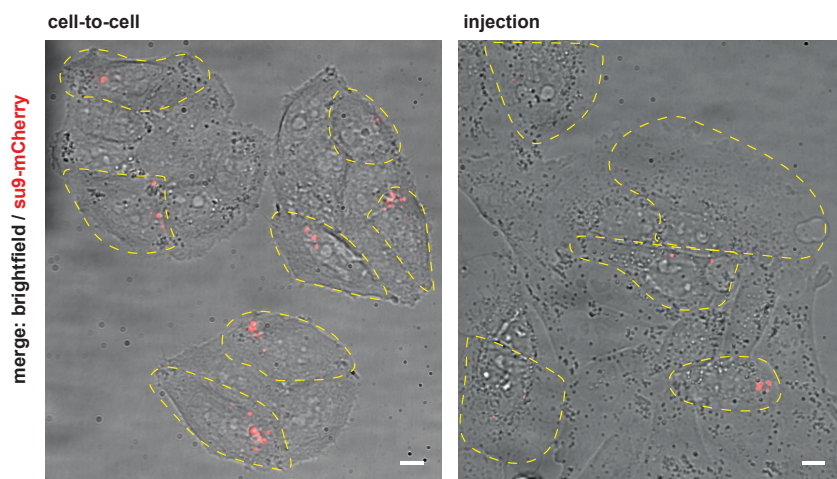

B

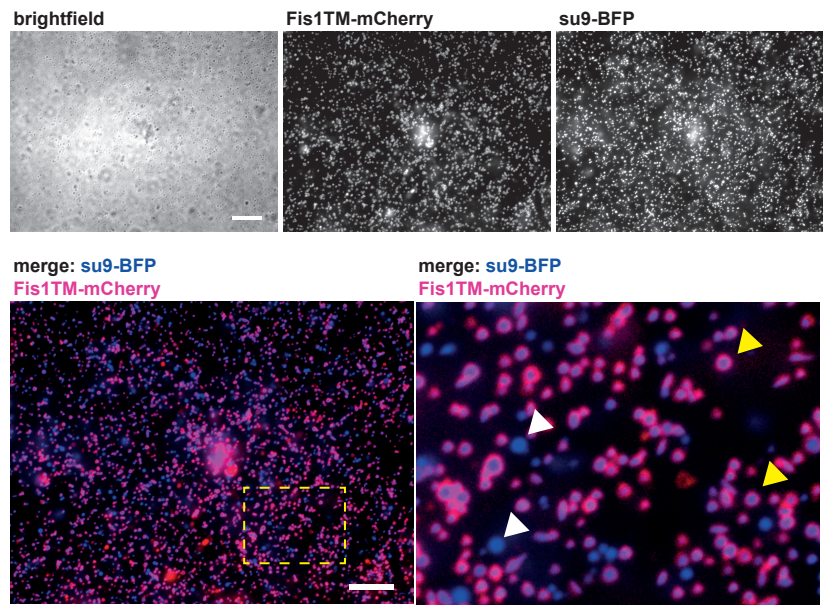

Supplement: S7 Fig — (A) Images of Hela cells postmitochondrial transplantations via the cell-to-cell and the injection approach. Images show and overlay of brightfield (gray) and the transplant (su9-mCherry, red). Transplanted cells are outlined in yellow. (B) Fluorescence-microscopy images of bulk-extracted mitochondria with labeled matrix (su9-BFP, blue) and labeled outer membrane (Fis1TM-mCherry, red). Yellow arrows indicate mitochondrial particles showing both, matrix and outer membrane labels. White arrows indicate mitochondrial particles missing the outer membrane. Scale bars: 10 μm. (PDF) [file pbio.3001576.s007.pdf]

**A**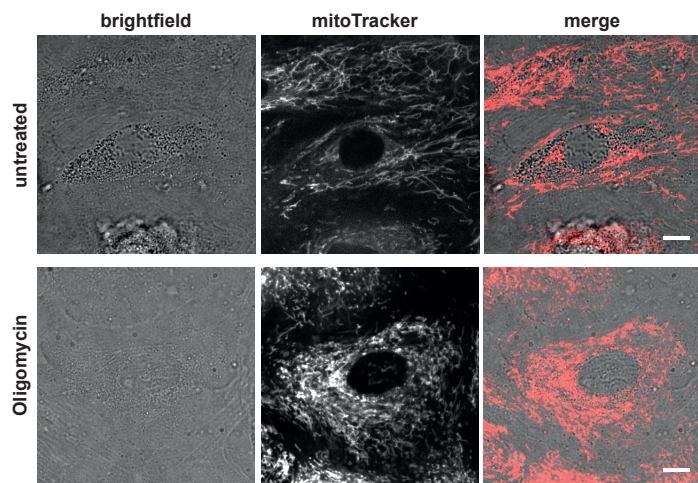**B**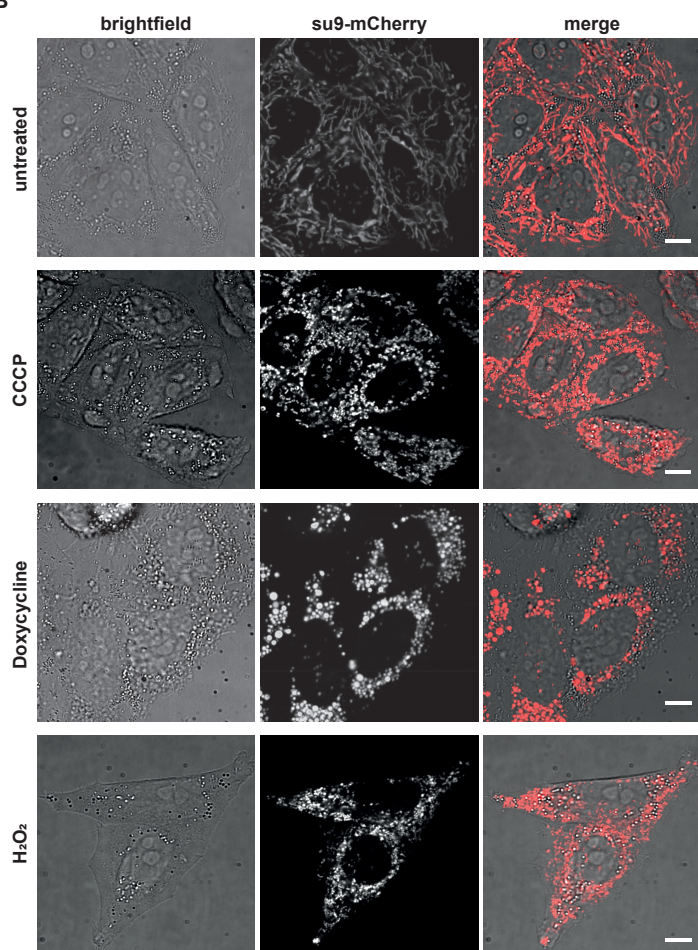

Supplement: S8 Fig — Fusion states of the mitochondrial network of HEKa cells upon drug-treatments. (A) HEKa cells in culture medium, the mitochondrial network is visualized using mitoTracker Green. Cells in the lower panel were treated with 4 μM oligomycin for 22 hours. (B) Hela cells in culture medium, the mitochondrial matrix is visualized via permanent expression of su9-mCherry. Treatments: 10 μM CCCP for 3 hours, 8 μM doxycycline for 24 hours, 750 μM H2O2 for 3 hours. Scale bars: 10 μm. HEKa, human endothelial keratinocyte. (PDF) [file pbio.3001576.s008.pdf]

**a**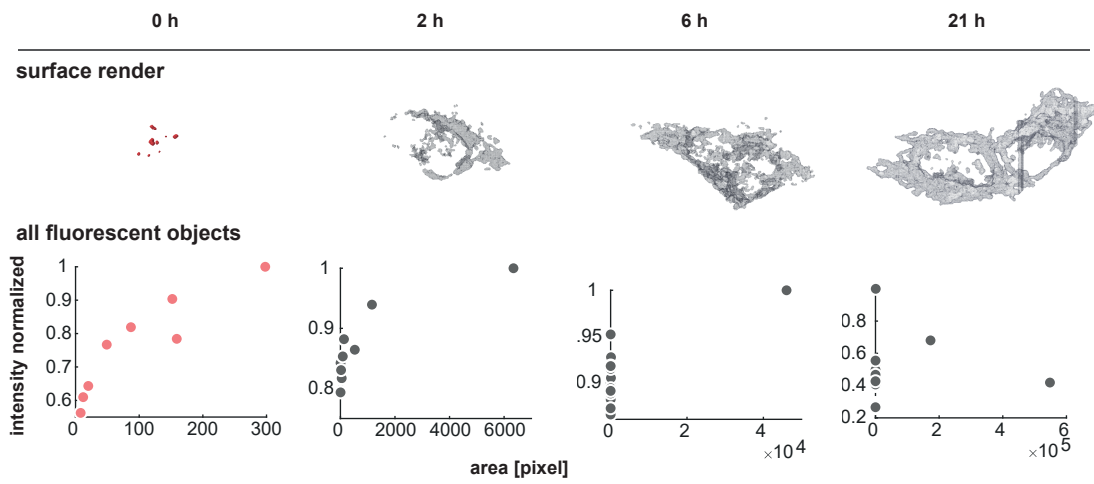**b**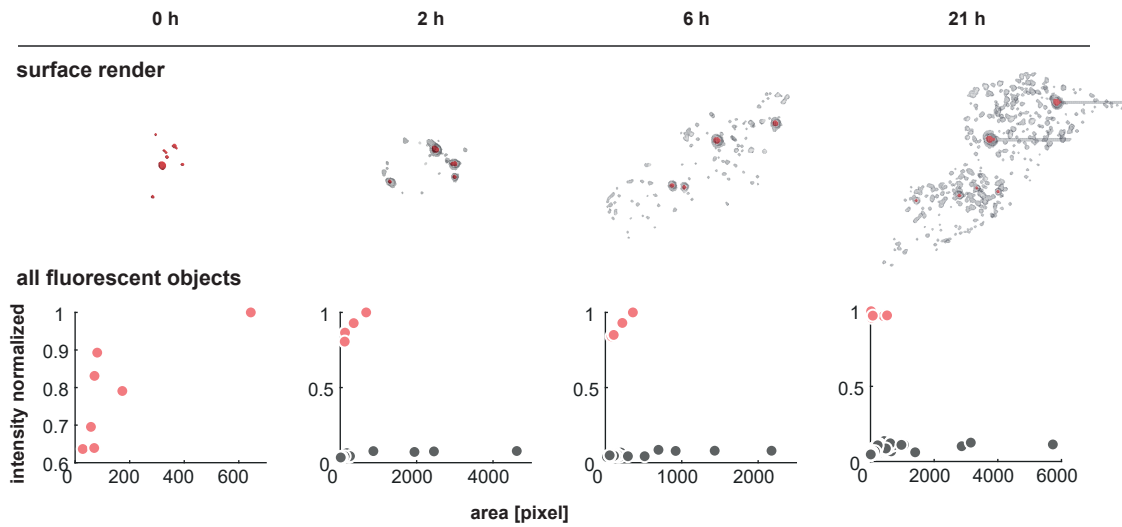

Supplement: S9 Fig — Visualization and analysis of transplanted mitochondria in single Hela cells. Top: surface render of the total fluorescence of the transplant over time bottom: objects plotted by size in pixel and normalized fluorescence intensity. Objects carrying the high fluorescence intensity of the initial transplant at the 0 hour time point are depicted in red; objects with low fluorescence are depicted in gray. (A) Hela cell showing mitochondrial acceptance of 8 mitochondria within 2 hours. (B) Hela cell showing full degradation of the transplant, 7 mitochondria were transplanted. The data underlying S9A and S9B Fig can be found in S1 Data. (PDF) [file pbio.3001576.s009.pdf]

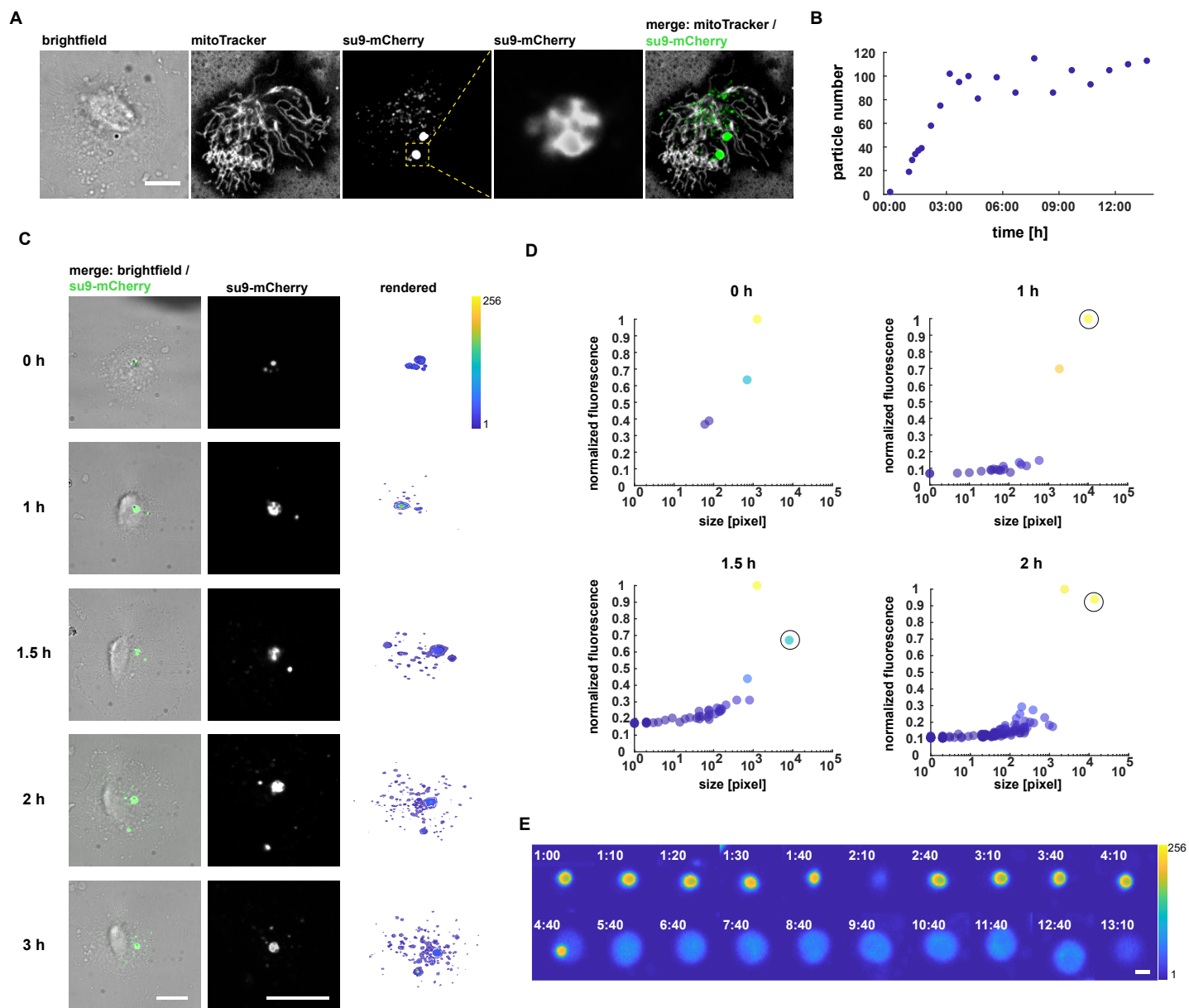

Supplement: S10 Fig — Exemplary quantification of mitochondrial degradation in HEKa cells. (A) Fluorescence microscopy images of a cell 19 hours postmitochondrial transplantation, 4 mitochondria were initially transplanted. The enlarged section shows a presumptive mitophagosomal structure. Scale bar: 10 μm. (B) Total number of fluorescent objects within the host cell over time. (C) Time-lapse images of mitochondrial degradation. Surface-rendered images of all detected fluorescent images show the increasing number and spatial distribution of particles over time. Scale bar: 10 μm. (D) Scatter plots of objects plotted by size in pixel (logarithmic scale) and normalized fluorescence intensity. Outlined dot shows the largest tertiary particle shown in the central panel in c. e, degradation of individual mitochondria over time [hour]. After fusion with a presumptive mitophagosomal structure at 4:40 hours, the degradation progresses for over 8 hours. Scale bar: 1 μm. The data underlying S10B and S10D Fig can be found in S1 Data. HEKa, human endothelial keratinocyte. (PDF) [file pbio.3001576.s010.pdf]

A

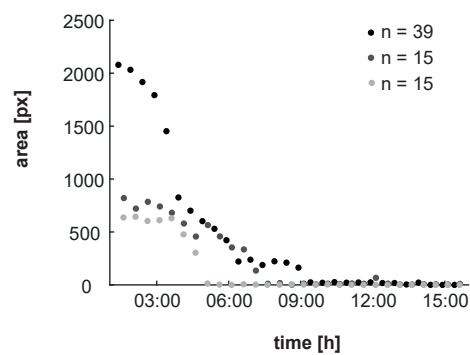

B

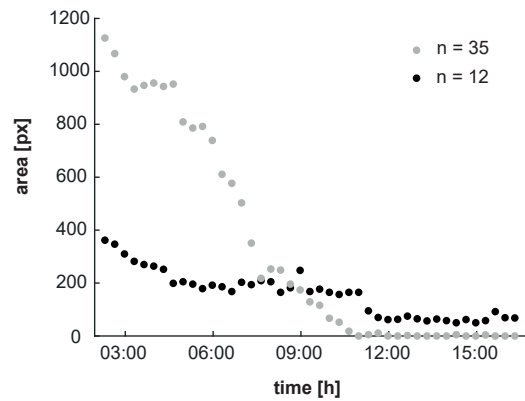

C

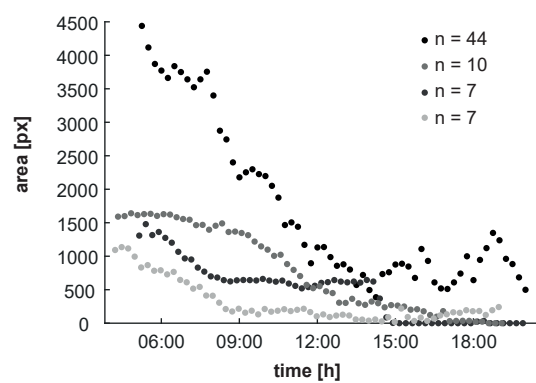

Supplement: S11 Fig — Fluorescent traces of the mitochondrial transplant and directly derived particles in single HEKa cells over time. Plots show the total volume occupied by unfused mitochondrial transplant over time in various conditions. The number of initially transplanted mitochondria per cell is depicted on the upper right. (A) Transplant was treated with CCCP and oligomycin. (B) Transplant was treated with doxycycline, CCCP, and oligomycin. (C) Transplant was treated with doxycycline. The data underlying S11A–S11C Fig can be found in S1 Data. HEKa, human endothelial keratinocyte. (PDF) [file pbio.3001576.s011.pdf]

A

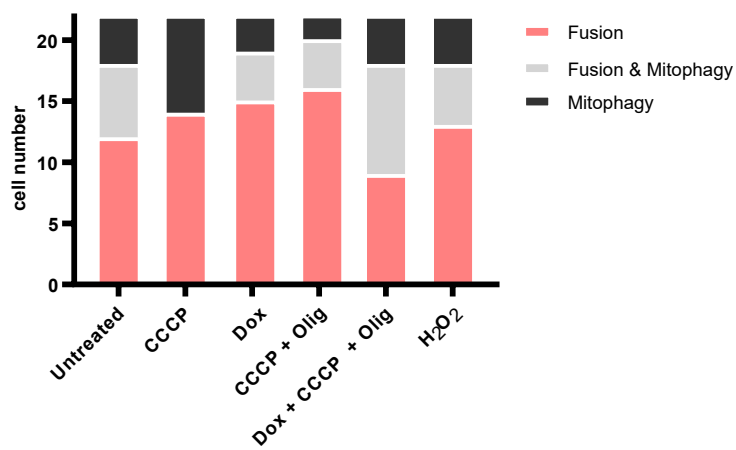

B

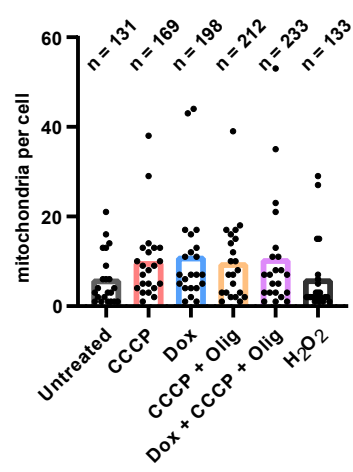

Supplement: S12 Fig — (A) Fusion and degradation behavior of drug-compromised mitochondrial transplants in HEKa cells. Each condition was tested with 22 cells. (B) Distribution of mitochondrial quantity transplanted per cell from all conditions tested in a (22 cells for each conditions). Line shows median value. The data underlying S12A and S12B Fig can be found in S1 Data. HEKa, human endothelial keratinocyte. (PDF) [file pbio.3001576.s012.pdf]

**a**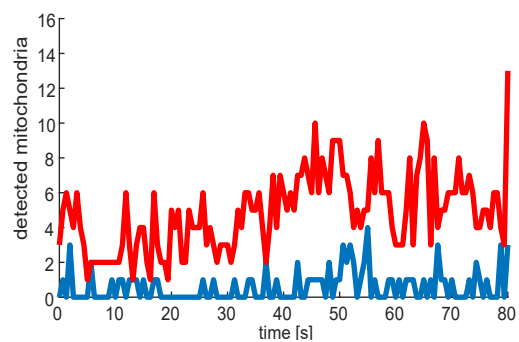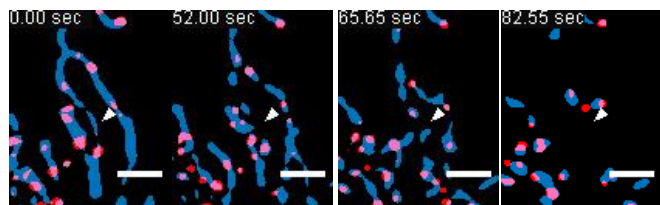**b**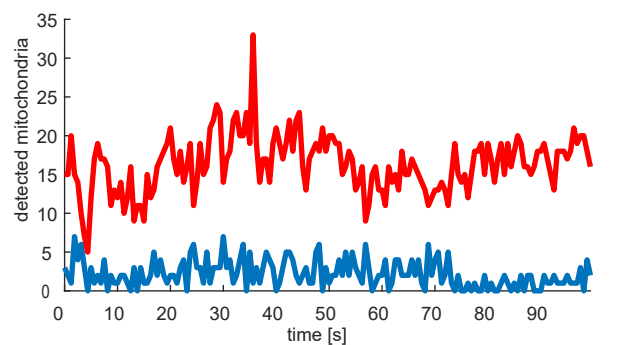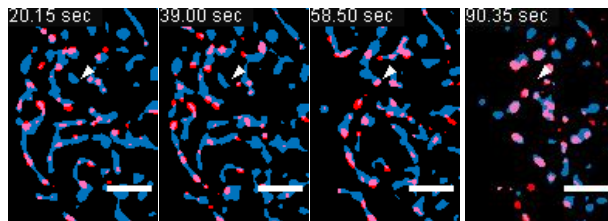**c**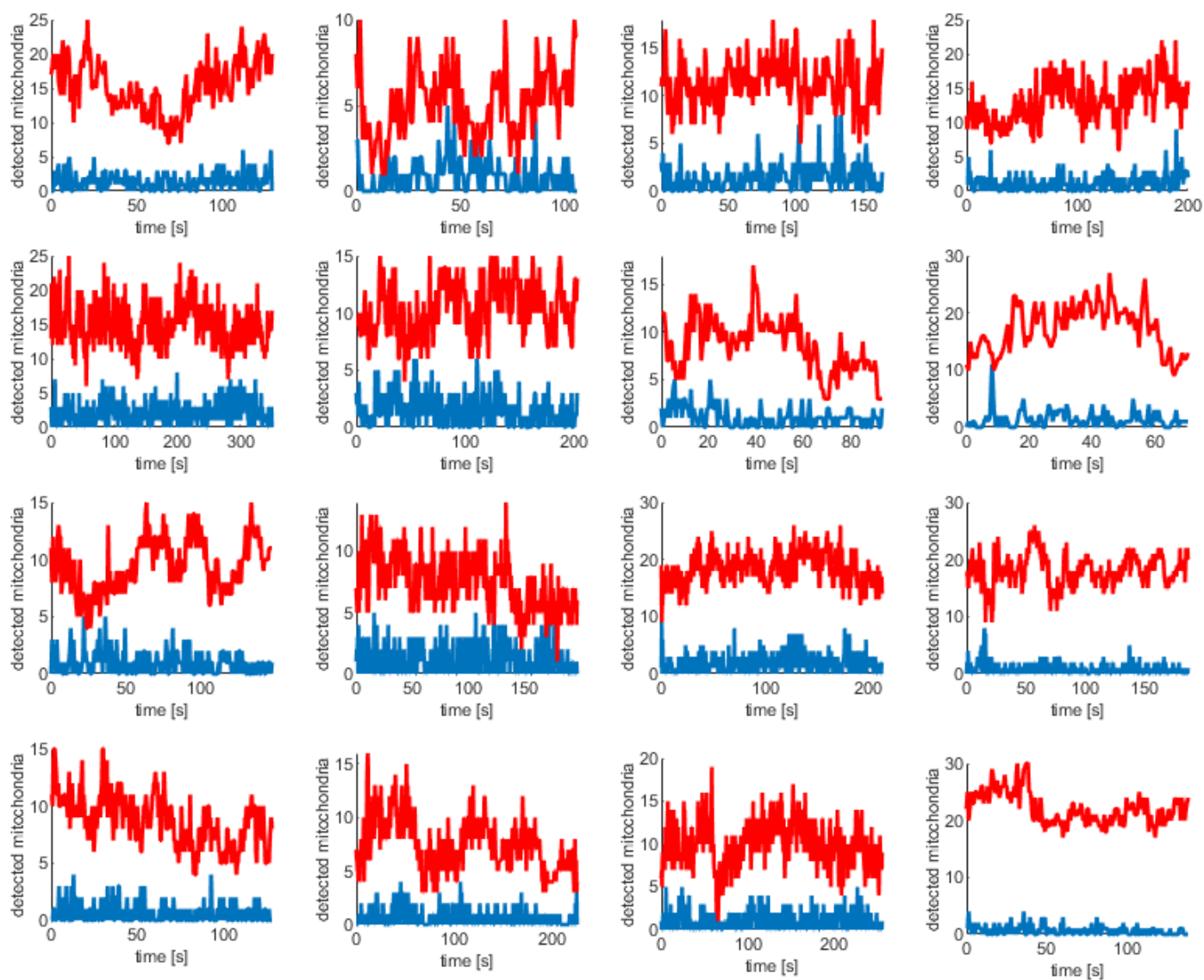

Supplement: S13 Fig — Tracing of p55 nucleoids overlap with mitochondria during extraction via FluidFM. (A, B) Traces of separated mitochondria (su9-BFP) showing overlap with p55-GFP signals in red and of separated mitochondria showing no traces of p55-GFP in blue. White arrowhead indicates cantilever apex position. Panels below show the extraction sites comprising the area used for data acquisition. The mitochondrial network fluorescence is shown in blue, p55-GFP speckles are shown in red. Scale bars: 5 μm. (C) Traces of all cells analyzed in this manner. The data underlying S13A–S13C Fig can be found in S1 Data. (PDF) [file pbio.3001576.s013.pdf]
