# Supplementary material for: Mitochondria transplantation between living cells
Source: PLoS Biol. 2022 Mar 23;20(3):e3001576. doi: 10.1371/journal.pbio.3001576 (PMC8942278; doi:10.1371/journal.pbio.3001576)
Supplement: S1 Text — (DOCX) [file pbio.3001576.s026.docx]

S1_Text

Force-induced mitochondrial fission

It has previously been suggested that mitochondrial membrane constriction is a prerequisite for mitochondria fission [1,2]; however it was impossible to exert highly localized hydrodynamic pulling forces intracellularly with sub micrometer resolution. FluidFM has the advantage of allowing to distinguish between mechanical force exertion and other cellular processes possibly involved such as calcium signaling. When extracting mitochondria, we observed induction of the pearls-on-a-string phenotype on mitochondria, followed by division of the inner-and outer mitochondrial membrane (S5A,B, Fig; S5 and S6 Movies). We wondered whether the scission process was due to mechanical forces exerted by FluidFM or by recruitment of the native mitochondrial fission machinery to these sites. A main component of this machinery is Drp1, a mechanoenzyme that assembles circularily around mitochondria and uses the energy from GTP hydrolysis to mediate membrane scission [3]. To assess the recruitment of the fission machinery to constricted sites, we expressed a fluorescently labelled version of Drp1 (Drp1-mCherry) in U2OS cells and performed mitochondrial pulling experiments to follow the fluorescent signals of both the mitochondrial matrix and Drp1. We observed that the FluidFM-induced pearls-on-a-string phenotype led to the recruitment of fluorescently labelled Drp1 (Drp1-mCherry) at the induced constriction sites of targeted mitochondrial tubes (S5D Fig, n = 18), thus providing a direct link between induced constriction and recruitment of Drp1.

The observed force-induced shape transition leads to the question of its relevance *in vivo*. To investigate this question further we examined kinesin as an endogenous motor protein [4] as a potential trigger. We employed the split-kinesin strategy using rapamycin-inducible protein interactions [5]. Briefly, FK506 binding protein (FKBP) was fused to mCherry and to the transmembrane domain of Fis1 for OMM targeting. Its partner, FKBP–rapamycin binding (FRB), was fused 20 to the motor domain of kinesin. Upon rapamycin exposure, FKBP and FRB form a stable complex, thereby coupling mitochondria to the kinesin motor domain and directs their transport on microtubules. Addition of rapamycin to cells expressing these constructs induced a global shape transition of mitochondria similar to that observed upon FluidFM aspiration (S5E Fig), suggesting that hydrodynamic pulling forces created by FluidFM aspiration are in a similar range as forces created by kinesin motor proteins.

These results are congruent with previous studies suggesting that mitochondrial membrane constriction is a prerequisite for mitochondria fission [1,2,6,7]. An indicator of plasma membrane damage and an inducer of mitochondrial pearling is leakage of calcium ions into the cytoplasm and mitochondria [8]. Mammalian cells tightly control calcium concentrations whereby the ER acts as Ca^2+^ storage compartment and mitochondrial pearling and subsequent fission has previously been associated with Ca^2+^ signaling [9,10]. To investigate whether Ca^2+^ flux is associated with shape transition of mitochondria in our approach, we used the Ca^2+^ sensitive fluorophore mito-R-GECO1 [11] targeted to the mitochondrial matrix and followed mitochondrial Ca^2+^ dynamics in time-lapse microscopy experiments. We observed no change in signal intensity of R-GECO1, neither after probe insertion, nor during the extraction process (S7 Movie). To control for functionality of the sensor system, we probed the initially extracted cells twice (n =17). In the second approach, we manually displaced the probe while it was inside the cell, inducing rupture of the cytoplasmic membrane. Because the cell culture medium contains roughly a 6000-fold excess of calcium compared to mitochondria [8], we expected an influx of Ca^2+^. Indeed, a systemic Ca^2+^ influx signal of mito-R-GECO occurred, propagating radially from the probe insertion site, followed by rounding of mitochondria and cell death (S8 Movie), in line with Ca^2+^ inducing apoptosis via cytochrome *c* release from mitochondria [8]. A similar mitochondrial calcium influx was observed when using imperfectly coated probes that we expected to result in localized Ca^2+^ influx upon membrane puncture. Indeed, we observed a rapid and transient increase of R-GECO1 fluorescence intensity in mitochondria upon probe insertion. However, there was no immediate influence on the mitochondrial morphology, indicating that the Ca^2+^ threshold for permeability transition was not reached [12]. Only when negative pressure was applied, ‘pearling’ of mitochondrial tubes was observed exclusively in direct proximity of the aperture. The morphology of mitochondria situated further away did not change, despite being equally affected by the Ca^2+^ influx (n=14) (S6A Fig and S9 Movie). Finally, we depleted Ca^2+^ in the medium by adding the chelating agent EGTA. Under this condition, the signal intensity of R-GECO1 did not rise, neither upon probe entry nor during the mechanically induced fission process (S6B Fig and S10 Movie). To rule out an involvement of calcium stored within the ER, we treated U2OS-cells with thapsigargin, which depletes the ER calcium reservoir. We did not detect any impact of thapsigargin on force induced pearling of mitochondria (S6C Fig). Based on these results, we conclude that calcium influx is not linked to the observed mitochondrial shape transition. Due to the directional propagation along mitochondrial tubes, rather than a radial propagation from the site of probe insertion, as well as lacking evidence of an influence of calcium on the observed process, we conclude that hydrodynamic pulling forces rather than a biochemical signal is responsible for mitochondrial pearling and fission.

1. Helle SCJ, Feng Q, Aebersold MJ, Hirt L, Grüter RR, Vahid A, et al. Mechanical force induces mitochondrial fission. Elife. 2017;6: 1–26. doi:10.7554/eLife.30292

2. Mahecic D, Carlini L, Kleele T, Colom A, Goujon A, Matile S, et al. Mitochondrial membrane tension governs fission. Cell Rep. 2021;35. doi:10.1016/j.celrep.2021.108947

3. Smirnova E, Shurland DL, Ryazantsev SN, Van Der Bliek AM. A human dynamin-related protein controls the distribution of mitochondria. J Cell Biol. 1998;143: 351–358. doi:10.1083/jcb.143.2.351

4. Meyhofer E, Howard J. The force generated by a single kinesin molecule against an elastic load. Proc Natl Acad Sci. 1995;92: 574–578. doi:10.1073/pnas.92.2.574

5. van Bergeijk P, Hoogenraad CC, Kapitein LC. Right Time, Right Place: Probing the Functions of Organelle Positioning. Trends Cell Biol. 2016;26: 121–134. doi:10.1016/j.tcb.2015.10.001

6. Friedman JR, Lackner LL, West M, DiBenedetto JR, Nunnari J, Voeltz GK. ER tubules mark sites of mitochondrial division. Science (80- ). 2011;334: 358–362. doi:10.1126/science.1207385

7. Cho B, Cho HM, Jo Y, Kim HD, Song M, Moon C, et al. Constriction of the mitochondrial inner compartment is a priming event for mitochondrial division. Nat Commun. 2017;8: 15754. doi:10.1038/ncomms15754

8. Ghibelli L, Cerella C, Diederich M. The dual role of calcium as messenger and stressor in cell damage, death, and survival. Int J Cell Biol. 2010;2010. doi:10.1155/2010/546163

9. van der Bliek AM, Shen Q, Kawajiri S. Mechanisms of mitochondrial fission and fusion. Cold Spring Harb Perspect Biol. 2013;5. doi:10.1101/cshperspect.a011072

10. Nemani N, Carvalho E, Tomar D, Dong Z, Ketschek A, Breves SL, et al. MIRO-1 Determines Mitochondrial Shape Transition upon GPCR Activation and Ca2+ Stress. Cell Rep. 2018;23: 1005–1019. doi:10.1016/j.celrep.2018.03.098

11. Wu J, Liu L, Matsuda T, Zhao Y, Rebane A, Drobizhev M, et al. Improved orange and red Ca2+ indicators and photophysical considerations for optogenetic applications. ACS Chem Neurosci. 2013;4: 963–972. doi:10.1021/cn400012b

12. Chaudhuri D, Artiga DJ, Abiria SA, Clapham DE. Mitochondrial calcium uniporter regulator 1 (MCUR1) regulates the calcium threshold for the mitochondrial permeability transition. Proc Natl Acad Sci U S A. 2016;113: E1872–E1880. doi:10.1073/pnas.1602264113
