# Supplementary material for: Mitochondria transplantation between living cells
Source: PLoS Biol. 2022 Mar 23;20(3):e3001576. doi: 10.1371/journal.pbio.3001576 (PMC8942278; doi:10.1371/journal.pbio.3001576)
Supplement: S1 Table — (PDF) [file pbio.3001576.s028.pdf]

Supplementary tables:

Supplementary Table 1. Viability of HeLa cells post mitochondrial transplantation: Injection of purified mitochondria, extracted from bulk cultured cells

Control - PCR-amplified U2OS mtDNA was mixed with PCR-amplified HeLa mtDNA to the following concentrations: Ctl1 - 0.1 % / Ctl2 - 0.5 % / Ctl3 - 1 %

**position**	**Ctl1.A**	**Ctl1.C**	**Ctl1.G**	**Ctl1.T**	**Total reads**	**HeLa mtDNA [%]**	**U2OS mtDNA [%]**	**average [%]**
15959	3	0	20059	18	20080	99.90	0.09	0.10
16069	0	19831	0	20	19851	99.90	0.10	
16108	0	19650	0	20	19670	99.90	0.10	
16126	0	19	0	19639	19658	99.90	0.10	
**position**	**Ctl2.A**	**Ctl2.C**	**Ctl2.G**	**Ctl2.T**	**Total reads**	**HeLa mtDNA [%]**	**U2OS mtDNA [%]**	**average [%]**
15959	5	0	28675	120	28800	99.57	0.42	0.47
16069	1	28388	0	141	28530	99.50	0.49	
16108	0	28139	0	140	28279	99.50	0.50	
16126	0	137	0	28129	28266	99.52	0.48	
**position**	**Ctl3.A**	**Ctl3.C**	**Ctl3.G**	**Ctl3.T**	**Total reads**	**HeLa mtDNA [%]**	**U2OS mtDNA [%]**	**average [%]**
15959	2	0	25492	164	25658	99.35	0.64	0.77
16069	1	25226	0	210	25437	99.17	0.83	
16108	1	25021	0	210	25232	99.16	0.83	
16126	0	201	0	25016	25217	99.20	0.80	

Transplanted - biological replicates 1-3 (i)

position	TP1. A	TP1. C	TP1. G	TP1. T	Total reads	HeLa mtDNA [%]	U2OS mtDNA [%]	average [%]
15959	0	0	27638	658	28296	97.67	2.33	2.58
16069	0	27360	0	757	28117	97.31	2.69	
16108	0	27296	0	750	28046	97.33	2.67	
16126	0	734	0	27263	27997	97.38	2.62	
**position**	**TP2. A**	**TP2. C**	**TP2. G**	**TP2. T**	**Total reads**	**HeLa mtDNA [%]**	**U2OS mtDNA [%]**	**average [%]**
15959	1	0	32063	498	32562	98.47	1.53	1.70
16069	1	31738	0	571	32310	98.23	1.77	
16108	0	31674	0	571	32245	98.23	1.77	
16126	0	560	0	31662	32222	98.26	1.74	
**position**	**TP3. A**	**TP3. C**	**TP3. G**	**TP3. T**	**Total reads**	**HeLa mtDNA [%]**	**U2OS mtDNA [%]**	**average [%]**
15959	0	0	15191	221	15412	98.57	1.43	1.58
16069	0	15097	0	253	15350	98.35	1.65	
16108	1	15081	0	252	15334	98.35	1.64	
16126	0	247	0	15084	15331	98.39	1.61	

Injected mitochondria from bulk isolation - biological replicates 1-3 (ii)

position	Inj1.A	Inj1.C	Inj1.G	Inj1.T	Total reads	HeLa mtDNA [%]	U2OS mtDNA [%]	average [%]
15959	0	0	12555	79	12634	99.37	0.63	0.70
16069	0	12434	0	92	12526	99.27	0.73	
16108	2	12403	0	92	12497	99.25	0.74	
16126	1	88	0	12398	12487	99.29	0.70	
**position**	**Inj2.A**	**Inj2.C**	**Inj2.G**	**Inj2.T**	**Total reads**	**HeLa mtDNA [%]**	**U2OS mtDNA [%]**	**average [%]**
15959	0	0	23824	116	23940	99.52	0.48	0.56
16069	0	23609	0	137	23746	99.42	0.58	
16108	1	23567	1	140	23709	99.40	0.59	
16126	0	135	0	23569	23704	99.43	0.57	
**position**	**Inj3.A**	**Inj3.C**	**Inj3.G**	**Inj3.T**	**Total reads**	**HeLa mtDNA [%]**	**U2OS mtDNA [%]**	**average [%]**
15959	0	0	29343	35	29378	99.88	0.12	0.14
16069	0	29123	0	44	29167	99.85	0.15	
16108	2	29080	0	45	29127	99.84	0.15	
16126	0	42	0	29075	29117	99.86	0.14	

Mixed - biological replicates 1-3 (iii)

**position**	**Mixed1.A**	**Mixed 1.C**	**Mixed.G**	**Mixed1.T**	**Total reads**	**HeLa mtDNA [%]**	**U2OS mtDNA [%]**	**average [%]**
15959	2	0	38482	3	38487	99.99	0.01	0.00
16069	0	38329	0	1	38330	100.00	0.00	
16108	0	38281	0	0	38281	100.00	0.00	
16126	1	0	0	38265	38266	100.00	0.00	
**position**	**Mixed 2.A**	**Mixed 2.C**	**Mixed.G**	**Mixed2.T**	**Total reads**	**HeLa mtDNA [%]**	**U2OS mtDNA [%]**	**average [%]**
15959	2	0	32911	0	32913	99.99	0.00	0.00
16069	0	32764	0	1	32765	100.00	0.00	
16108	1	32725	0	1	32727	99.99	0.00	
16126	0	0	0	32719	32719	100.00	0.00	
**position**	**Mixed 3.A**	**Mixed 3.C**	**Mixed3.G**	**Mixed3.T**	**Total reads**	**HeLa mtDNA [%]**	**U2OS mtDNA [%]**	**average [%]**
15959	3	0	38647	0	38650	99.99	0.00	0.00
16069	0	38446	0	0	38446	100.00	0.00	
16108	0	38402	0	1	38403	100.00	0.00	
16126	0	0	0	38389	38389	100.00	0.00	
