# Supplementary material for: Mitochondria transplantation between living cells
Source: PLoS Biol. 2022 Mar 23;20(3):e3001576. doi: 10.1371/journal.pbio.3001576 (PMC8942278; doi:10.1371/journal.pbio.3001576)
Supplement: S2 Table — (PDF) [file pbio.3001576.s029.pdf]

Supplementary Table 2. Primers used in this study

Name	Binding site mtDNA	Sequence 5' – 3'
Primer 1	15720	ATTGACTCCTAGCCGCAGAC
Primer 2	16298	AAGGGTGGGTAGGTTTGTTG
